# Quercetin in bone regeneration: optimization of drug delivery system and study of its synergistic mechanism with bone tissue engineering

**DOI:** 10.3389/fphar.2026.1776263

**Published:** 2026-06-24

**Authors:** Shunzhi Yang, Dingming Li, Mengyao Liu, Jiabin Song, Jinyao Li, Lu Sun, Ying Yang, Yunke Zhang, Min Zhao, Yanchen Feng, Feixiang Liu

**Affiliations:** 1 The First Affiliated Hospital of Henan University of Traditional Chinese Medicine, Zhengzhou, China; 2 Fifth Clinical Medical College, Henan University of Traditional Chinese Medicine, Zhengzhou, China; 3 School of Acupuncture and Tuina, Henan University of Traditional Chinese Medicine, Zhengzhou, China; 4 School of Traditional Chinese Medicine (Zhongjing College), Henan University of Traditional Chinese Medicine, Zhengzhou, China; 5 Henan Provincial Collaborative Innovation Center of Chinese and Western Medicine for Prevention and Treatment of Major Diseases, Zhengzhou, China

**Keywords:** bone marrow mesenchymal stem cells, bone tissue engineering, drug delivery systems, osteoblasts, osteoclasts, quercetin

## Abstract

Skeletal diseases impose a heavy global burden, creating an urgent demand for safe and effective therapeutic strategies for bone regeneration. Quercetin, a natural flavonoid compound, has attracted considerable attention owing to its potential to promote bone formation and inhibit bone resorption. Nevertheless, its poor solubility and low bioavailability limit its clinical application. The integration of drug delivery systems with bone tissue engineering offers a promising approach to address these challenges. This review examines the latest advances in quercetin-based drug delivery systems and their synergistic mechanisms with bone tissue engineering. It systematically discusses various strategies, including hydrogels, hydroxyapatite, biofunctional scaffolds, phospholipid carriers, metal complexes, nanomaterials, and medicinal chemical modifications, and presents the core advantages of these approaches in achieving targeted delivery, controlled release, and enhanced bioactivity of quercetin. Furthermore, this review highlights the central role of these delivery systems in potentiating the pro-regenerative effects of quercetin. These advances not only provide practical solutions to overcome the inherent limitations of quercetin but also significantly broaden its application prospects in bone tissue engineering.

## 1 Introduction

The skeletal system plays a vital role in supporting the body’s structure and facilitating motor function. Bone-related diseases, such as fractures, osteoarthritis, osteoporosis, bone defects, osteomyelitis, and bone tumors, are becoming increasingly prevalent with the aging global population and changes in modern lifestyles. According to the Institute for Health Metrics and Evaluation at Washington University School of Medicine, approximately 600 million people worldwide suffer from osteoarthritis, reflecting a 137% increase since 1990. Additionally, about 455 million people are affected by acute or chronic fractures (GBD, 2019 Fracture Collaborators, 2021). These bone diseases often lead to functional limitations, sleep disorders, disability, depression, and other symptoms, imposing a significant burden on both patients and society, and severely affecting quality of life (Parmelee et al., 2015; Weng et al., 2024; Yan et al., 2023). Following bone injury, the body typically progresses through four stages: inflammation, chondrogenesis, sclerotization, and remodeling, gradually restoring bone structure and function through the coordinated action of fibroblasts, chondrocytes, and osteoblasts (OBs), a process known as bone regeneration (Liu Y. L. et al., 2024; Kenkre and Bassett, 2018). Therefore, the ability to regenerate bone is crucial for the recovery of patients with bone diseases (Wang et al., 2021a). In complex clinical conditions such as osteoarthritis and osteoporosis, the body’s regenerative capacity is often insufficient for self-repair, and pharmacologic interventions are frequently required to maintain bone mass and slow bone loss. However, these drugs are often associated with adverse effects: for example, osteoporosis treatments like zoledronic acid and raloxifene may cause gastrointestinal discomfort, nephrotoxicity, and thrombotic risks (Dalle Carbonare et al., 2010; Messalli and Scaffa, 2010); osteoarthritis medications, such as meloxicam and etoricoxib, while effective in relieving pain and inflammation, can cause gastrointestinal, hepatic, renal, and cardiovascular toxicity (Richard et al., 2023); and methotrexate, used in the treatment of osteosarcoma, may cause renal tubular deposition when combined with chemotherapy (Holmboe et al., 2012). Additionally, bone grafting is often required for patients with severe bone defects or necrosis; however, the limited availability of autologous bone and the issue of allogeneic bone rejection still hinder its widespread application (Wojda and Donahue, 2018).

To overcome the limitations of bone regeneration and repair, bone tissue engineering has emerged as a promising solution. This field integrates the principles of biology and engineering, aiming to develop biological substitutes that can repair, maintain, and enhance the function of damaged bone tissue. The key steps involved include the acquisition and cultivation of seed cells, selection and preparation of scaffold materials, application of growth factors, and the construction of tissue-engineered bone *in vitro*. Ultimately, the resulting bone tissue complex is implanted into the patient, where it interacts with host tissues to promote bone formation and repair (Black et al., 2015; Zhang et al., 2020; Battafarano et al., 2021). Bone tissue engineering has become a focal point of medical research in recent years due to its significant potential in treating bone injuries and repairing bone defects (Manzini et al., 2021). However, bone morphogenetic proteins (BMPs) used in bone tissue engineering are costly and challenging to apply on a large scale, and they carry the risk of adverse effects such as ectopic osteogenesis, infection, inflammation, and carcinogenesis (Oryan et al., 2014). Therefore, the search for more effective, economical, and safer alternatives has become a critical research priority.

Quercetin (QUE) is a natural flavonoid compound with the chemical name 2-(3,4-dihydroxyphenyl)-3,5,7-trihydroxy-4H-chromen-4-one, a molecular formula of C_15_H_10_O_7_, and a molecular weight of 302.24. First isolated by Hungarian physiologist Albert Szent-Györgyi in 1936, QUE is widely found as glycosides in various fruits, vegetables, nuts, and herbs. It possesses multiple pharmacological activities, including anti-inflammatory, antioxidant, anticancer, antiviral, lipid-lowering, and hypoglycemic effects. Existing *in vitro* cell experiments and animal models have demonstrated that quercetin can enhance OB function, inhibits osteoclast (OC) activity, and promotes the proliferation of bone marrow mesenchymal stem cells (BMSCs) (Wang et al., 2022; Preethi and Bellare, 2021). Notably, QUE is considered safe, and large-scale high-purity production has been achieved using existing techniques such as solvent extraction, supercritical fluid extraction, and membrane separation (Pilařová et al., 2022; Kryvshenko et al., 2012). However, its poor water solubility, significant first-pass effect, and instability under physiological conditions limit its *in vivo* absorption and bioavailability (Sah et al., 2023). Recent studies have shown that bone tissue engineering, by simulating the three-dimensional *in vivo* microenvironment, can efficiently and directionally enhance the biological activity of QUE through multidimensional synergy. This paper systematically explores the role of drug delivery systems in improving the therapeutic efficacy of QUE and examines the close connection between QUE and bone tissue engineering, aiming to provide a theoretical foundation for its clinical application in bone tissue engineering. Given the instability of quercetin in aqueous solutions and under light, high-quality *in vivo* studies were performed with protection from light, fresh preparation, or drug delivery systems to ensure its bioactivity.

## 2 Mechanisms for promoting bone regeneration

**FIGURE 1:**
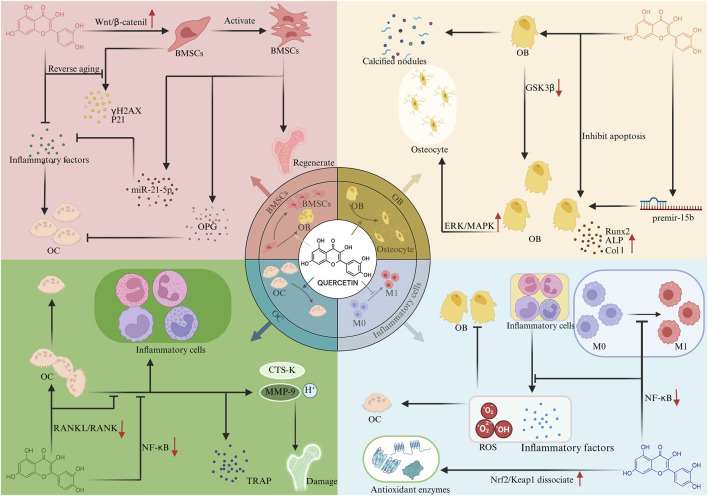
Schematic illustration of the multitarget regulatory mechanisms of quercetin in bone metabolism. Upper left quadrant: QUE activates the Wnt/β-catenin pathway to stimulate the proliferation and activation of BMSCs, promoting the transfer of miR-21-5p, which subsequently reduces inflammation factor levels and inhibits OC activity. QUE reduces the levels of P21 protein and γH2AX in aged BMSCs, while upregulating OPG to inhibit OC generation. Upper right quadrant: QUE promotes OB mineralization, inhibits OB apoptosis, and enhances the expression levels of ALP, Col I, and OB-specific miRNA (pre-miR-15b). By activating the ERK/MAPK pathway, QUE promotes OB differentiation and inhibits GSK3β, thereby increasing Runx2 expression. Lower left quadrant: QUE inhibits OC generation through the suppression of the RANKL/RANK signaling pathway and the NF-κB signaling pathway. Through multiple pathways, QUE decreases the levels of H+, MMP-9, CTS-K, and TRAP, thereby reducing the activation of inflammatory cells and preventing the degradation of bone matrix. Lower right quadrant: QUE reduces macrophage polarization to the M1 phenotype by inhibiting the NF-κB pathway, lowering the production of inflammatory cytokines and ROS, and reversing the inhibitory effects of inflammation factors and ROS on OBs, as well as the activation of OCs. Additionally, QUE enhances the dissociation of Nrf2 from Keap1, promoting the expression of endogenous antioxidant enzymes. Abbreviations: BMSCs: Bone Marrow Mesenchymal Stem Cells; OC: Osteoclast; OB: Osteoblast; OPG: Osteoprotegerin; ALP: Alkaline Phosphatase; Col I: Collagen Type I; RANKL: Nuclear Factor κB Receptor-Activated Ligand; RANK: Receptor Activator of Nuclear Factor - κB Receptor; CTS-K: Cathepsin K; MMP: Matrix Metalloproteinase; TRAP: Tartrate-Resistant Acid Phosphatase; ROS: Reactive Oxygen Species; NF-κB: Nuclear Factor-κB; Nrf2: Nuclear Factor Erythroid 2-Related Factor 2; Keap1: Kelch-Like ECH-Associated Protein 1; P21: Protein-Dependent Kinase Inhibitor 1A; γH2AX: DNA Damage Marker Phosphorylated Histone H2AX; GSK-3β: Glycogen Synthase Kinase-3β; ERK: Extracellularly Regulated Kinase; MAPK: Mitogen-Activated Protein Kinase; Wnt: Wingless/Integrated.

QUE promotes bone regeneration mainly through a synergistic regulatory network, which enhances OB activity, inhibits OC-mediated bone resorption, and ameliorates inflammatory and oxidative stress status. As shown in Figure 1, QUE acts on four key cell types: OB, BMSCs, OC and inflammatory cells, accompanied by the regulation of core signaling pathways.

### 2.1 Regulation of bone formation-related cells

#### 2.1.1 Osteoblasts

OB is derived from the differentiation of BMSCs and serves as the core functional cell in bone formation (Wang et al., 2012). Its differentiation activity and functional status directly determine the construction and repair of bone tissue (Tsao et al., 2017). By secreting bone matrix proteins such as type I collagen (Col I) and osteocalcin (OCN), OB regulates calcium and phosphorus metabolism and promotes hydroxyapatite (HAP) deposition, ultimately constructing and strengthening bone tissue (Tsao et al., 2017).

QUE exerts a clear positive regulatory effect on OB and can comprehensively enhance osteoblast function (Bose et al., 2024). The key to QUE-mediated bone regeneration lies in the overall enhancement of OB activity at the cellular behavioral level: on the one hand, it promotes OB proliferation and migration, accelerating their local recruitment (Bose et al., 2024; Dong R. et al., 2024); on the other hand, it inhibits OB apoptosis and excessive autophagy (Dong R. et al., 2024; Xiong et al., 2023), maintaining cell survival and functional stability in the pathological microenvironment (Lezcano et al., 2022). Meanwhile, QUE enhances OB differentiation and matrix secretion, promoting the formation of mineralized nodules (Bose et al., 2024; Dong R. et al., 2024). At the molecular level, QUE uniformly upregulates the expression levels of core osteogenic markers such as osteoblast-specific transcription factor 2 (Runx2), alkaline phosphatase (ALP), and Col I thus laying the foundation for bone matrix synthesis (Raj Preeth et al., 2021; Vimalraj et al., 2018; Pandit et al., 2020). Regarding molecular regulatory mechanisms, the above biological effects rely on the synergistic activation of multiple core osteogenic pathways by QUE. It inhibits glycogen synthase kinase 3 beta (GSK-3β) and stabilizes β-catenin signaling, which represents a key step in promoting the expression of Runx2 and the osteoblast-specific transcription factor Osterix (Osx) (Lezcano et al., 2022; Feng et al., 2024); meanwhile, it activates the extracellular regulated protein kinases (ERK)/mitogen-activated protein kinase (MAPK) signaling pathway to enhance cell proliferation and differentiation (Wang et al., 2014); it also mediates the activation of the BMP2/Smad pathway via the estrogen receptor (ER), thereby driving the synthesis of bone matrix proteins (Wong et al., 2020). These pathways act synergistically to ultimately enhance OB activity and promote bone matrix mineralization (Feng et al., 2024). Furthermore, the regulatory effect of QUE on osteogenesis has also been globally demonstrated in animal pathological models. QUE can significantly ameliorate the suppressed OB function in rats with abnormal bone metabolism, improve bone mineral density, bone mass and trabecular bone (Tb) parameters, and correct disorders of calcium and phosphorus metabolism, thereby alleviating insufficient bone formation at the organismal level (Pandit et al., 2020; Xiong et al., 2023).

In summary, with OB as its core target, QUE effectively enhances bone formation capacity by promoting differentiation and mineralization while inhibiting apoptosis, thereby exerting positive regulation on bone genesis, which is crucial for its bone regeneration-promoting effect (Figure 1).

#### 2.1.2 Bone marrow mesenchymal stem cells

BMSCs are multipotent adult stem cells that maintain bone homeostasis and serve as the core cells responsible for initiating osteogenic differentiation during bone regeneration. They can differentiate directionally into OB, mediate bone matrix synthesis and mineralized deposition (Chen et al., 2023), and participate in regulating the receptor activator of nuclear factor κB ligand (RANKL)/osteoprotegerin (OPG) balance, thereby modulating bone metabolic homeostasis (Chen et al., 2023). In the fracture microenvironment, BMSCs also reduce the levels of inflammatory factors such as tumor necrosis factor alpha (TNF-α), promoting the progression of bone regeneration (Dong S. et al., 2024).

Another important pathway through which QUE promotes bone regeneration is by comprehensively enhancing the osteogenic potential and survival ability of BMSCs (Gupta et al., 2017). It can significantly increase the proliferative activity of BMSCs and drive their directional differentiation into the osteogenic lineage (Gupta et al., 2017). Meanwhile, it can selectively eliminate senescent BMSCs, improve cellular metabolic status (Zhou et al., 2021), and enhance the adaptability of BMSCs in injured and pathological environments. In addition, QUE can enhance the mineralization capacity of BMSCs, providing a structural basis for new bone formation (Bian et al., 2021). In the molecular regulatory network, QUE coordinately regulates multiple signaling axes to achieve precise promotion of osteogenic differentiation in BMSCs. It modulates the H19/miR-625-5p axis to promote β-catenin accumulation and nuclear translocation, and activates BMP2/Smad signaling via ER-related pathways, enhancing transcriptional activation of osteogenesis-associated genes, thereby upregulating the levels of core osteogenic markers including Runx2, ALP and OCN (Feng et al., 2024; Bian et al., 2021; Srivastava et al., 2013). Meanwhile, QUE inhibits aberrant activation of repetitive elements and thereby blocks the RIG-I/TBK1-mediated RNA sensing pathway, reducing the expression levels of the senescence marker cyclin-dependent kinase inhibitor 1a (P21) and the DNA damage marker phosphorylated histone H2AX (γH2AX) in senescent BMSCs, ultimately optimizing the bone microenvironment and improving the osteogenic efficiency of BMSCs (Sun Y. et al., 2025). Furthermore, in animal models of bone defects, QUE can effectively upregulate the expression of osteogenic genes including OCN and Col I, induce the differentiation of BMSCs into OB (Song et al., 2018), and significantly increase the area of new bone formation (Zhou et al., 2021), verifying its pro-osteogenic effect *in vivo*.

In summary, targeting BMSCs, QUE significantly enhances bone regeneration by promoting proliferation, driving osteogenic differentiation, and ameliorating cellular senescence, which represents an important mechanism underlying its osteoprotective and pro-repair effects (Figure 1).

### 2.2 Regulation of osteoclasts

OC is the core functional cell mediating bone resorption. Derived from the differentiation and maturation of the monocyte-macrophage lineage, it accomplishes bone resorption and remodeling via the secretion of protons and proteolytic enzymes such as matrix metalloproteinase (MMP) and cathepsin K (CTSK) (Kodama and Kaito, 2020). Under physiological conditions, OC and OB coordinately maintain bone metabolic homeostasis (Cheng et al., 2024). However, overactive OC leads to bone loss and aggravated bone destruction, representing a key pathological process in bone defects, osteoporosis and other related diseases (Cheng et al., 2024).

Studies have shown that an important pathway mediating the osteoprotective effect of QUE is the inhibition of excessive OC activation and bone resorption (Guo et al., 2012). It markedly suppresses the differentiation and maturation of OC precursor cells, directly inhibits bone resorption activity, and promotes OC apoptosis, thereby blocking pathological bone resorption at the cellular level (Guo et al., 2012). Molecular mechanistic studies have demonstrated that the core mechanism underlying the potent anti-resorptive effect of QUE lies in its ability to effectively inhibit the RANKL/receptor activator of nuclear factor κB (RANK) signaling pathway and the expression of its key downstream transcription factor, nuclear factor of activated T cells cytoplasmic 1 (NFATc1) (Feng et al., 2024; Guo et al., 2012; Cheng et al., 2010). This consequently reduces the levels of bone resorption-related factors including CTSK, MMP-9, and tartrate-resistant acid phosphatase (TRAP) (Cheng et al., 2010; Forte et al., 2016; Masuhara et al,. 2016), while downregulating the expression of tumor necrosis factor receptor-associated factor 6 and cyclooxygenase-2 (Guo et al., 2012). In addition, QUE prevents the degradation of nuclear factor κB inhibitor (IκB) and the nuclear translocation of nuclear factor κB (NF-κB), thereby blocking the activation of the NF-κB signaling pathway (Yamaguchi and Weitzmann, 2011). It also inhibits the activation of the p38/MAPK and JNK/MAPK signaling pathways downstream of RANKL (Feng et al., 2024), thereby synergistically suppressing the differentiation and maturation of OC precursors as well as their bone resorptive activity through multiple pathways (Feng et al., 2024; Yamaguchi and Weitzmann, 2011). The regulatory effects described above at the cellular and molecular levels have been further validated in in vivo experiments. In animal models of bone erosion, QUE significantly reduces OC number and bone resorption activity, alleviates alveolar and joint bone destruction, and improves the osteoimmune microenvironment, thereby maintaining bone homeostasis at the systemic level (Taskan and Gevrek, 2020; Zhou et al., 2023).

In summary, by inhibiting OC differentiation, blocking core activation pathways, and balancing the RANKL/OPG system, QUE effectively suppresses pathological bone resorption, which constitutes an indispensable mechanism for its anti-bone-destruction and bone regeneration-promoting effects (Figure 1).

### 2.3 Anti-inflammatory and anti-oxidative stress

Inflammatory response and oxidative stress are important triggers of bone metabolic disorders and impaired bone regeneration (Haacke et al., 2025). In the bone injury microenvironment, inflammatory cells can release pro-inflammatory factors such as TNF-α and interleukin-1 beta (IL-1β), which in turn promote OC differentiation and maturation while inhibiting the osteogenic function of OB (Xu et al., 2023). Abnormally elevated levels of reactive oxygen species (ROS) in the microenvironment not only suppress OB proliferation and Col I synthesis but also induce osteocyte apoptosis (Domazetovic et al., 2017). Furthermore, inflammatory conditions and oxidative stress synergize to form a vicious cycle, ultimately aggravating bone loss and bone tissue destruction (Domazetovic et al., 2017).

The osteoprotective and bone regeneration-promoting effects of QUE are inseparable from its remarkable anti-inflammatory and anti-oxidative stress functions (Alharbi et al., 2025; Jafarbeglou et al., 2024). QUE can effectively reduce inflammatory cell infiltration (Jafarbeglou et al., 2024), inhibit macrophage polarization toward the pro-inflammatory phenotype (M1 type) (Comalada et al., 2005), reverse elevated inflammatory cytokine levels (Wang et al., 2023), and upregulate the activities of antioxidant enzymes (Farag et al., 2021; Messer et al., 2015), Meanwhile, QUE promotes mitochondrial biogenesis to ameliorate oxidative stress status (Shen et al., 2021). At the molecular level, QUE significantly downregulates the expression of various pro-inflammatory factors (such as IL-1α, IL-6, IL-1β, IL-8, and TNF-α) and oxidative stress markers (such as RANKL and ROS) (Napimoga et al., 2013; Forte et al., 2017), and reduces the levels of effectors associated with tissue damage induced by inflammation and oxidative stress (such as MMP-1 and MMP-3) (Rattanaprukskul et al., 2025). Further mechanistic studies reveal that QUE prevents the degradation of IκB and the nuclear translocation of the NF-κB p65 subunit (Han et al., 2023; Li et al., 2021), and significantly downregulates the expression of pro-inflammatory factors (such as TNF-α, IL-1β, IL-6) and M1-type markers (such as CD86) (Han et al., 2023; Mooney et al., 2021). Meanwhile, inhibition of the RANKL signaling pathway by QUE not only directly reduces inflammatory mediator levels but also indirectly blocks OC formation, thereby improving the inflammatory microenvironment of bone metabolism (Feng et al., 2024; Niu et al., 2020). In addition, the promotion of dissociation and nuclear translocation of nuclear factor erythroid 2-related factor 2 (Nrf2) from Kelch-like ECH-associated protein 1 (Keap1) is central to the antioxidant effect of QUE. This further activates the antioxidant response element and upregulates the expression of various endogenous antioxidant enzymes, including superoxide dismutase, heme oxygenase-1, and catalase (Feng et al., 2024). Furthermore, in mice with Collagen-Induced Arthritis (CIA), QUE significantly alleviates joint swelling and reduces the degree of bone erosion (Han et al., 2023). Meanwhile, it effectively decreases the clinical arthritis score and ankle thickness in CIA mice, and reduces synovial hyperplasia as well as bone destructio (Shen et al., 2021).

In summary, by activating antioxidant pathways, blocking inflammatory signals, and regulating the bone immune microenvironment, QUE exerts dual effects of anti-inflammation and anti-oxidative stress, improves bone metabolic disorders, and removes obstacles for bone regeneration. This is an important auxiliary mechanism for it to maintain bone homeostasis and promote bone repair (Figure 1).

## 3 Drug delivery systems

The drawbacks of QUE, such as poor water solubility, rapid *in vivo* metabolism, and short half-life, have severely limited its clinical application in bone regeneration. In recent years, with continuous advancements in drug technology, researchers have developed various delivery systems for QUE, which not only effectively overcome these limitations but also offer additional advantages, including precise targeting and long-term stability for QUE (Bonifácio et al., 2014; Unnikrishnan Meenakshi et al., 2024; Khatoon et al., 2022).

### 3.1 Hydrogel materials

Hydrogel is a flexible crosslinked polymer network based on hydrophilic macromolecular monomers, usually crosslinked from hydrophilic monomers or polymers. It possesses high hydration, tunable structure, biomaterial, and biodegradation properties, thus mimicking the hydration environment of biological tissues (Kapusta et al., 2023). Hydrogels are highly biocompatible, adaptable to body tissues, and can reduce immune responses while avoiding accumulation in the body. Additionally, the rich water content and reticular structure of hydrogels facilitate the dispersion and release of drugs in the aqueous phase, increasing their solubility and improving the bioavailability of poorly water-soluble drugs (Ho et al., 2022). Moreover, the void structure and cross-linking degree of the hydrogel can be adjusted to sequester the drug from physiological conditions (Kapusta et al., 2023). The rate of drug release can also be precisely controlled to maintain effective concentrations over an extended period (Farasati Far et al., 2024). These properties make hydrogels ideal bone replacement materials and show great potential in the field of bone regeneration.

Folic acid (FA) is a water-soluble B vitamin capable of mediating active targeting. FA-modified QUE microemulsions dispersed in a hydrogel matrix were found to possess *in situ* thermosensitive phase transition properties, which could trigger an *in situ* solution-gel transition in the organism for precise and effective drug release. This thermosensitive hydrogel system provides a physical scaffold for OBs, which can exert strong ROS scavenging ability and modulate macrophage polarization. After injection into the periodontal pockets of rats with periodontitis, it not only significantly reduced collagen degradation and OC activity but also reestablished helper T cell 17/regulatory T cell homeostasis *in vivo* (Li J. et al., 2024). Based on sensing the external environment, the microenvironment-responsive hydrogel, prepared using triglyceride monostearate, could also recognize a variety of MMPs, and therefore target senescent BMSCs more precisely to achieve local aggregation of QUE. After treatment with QUE-loaded microenvironment-responsive hydrogels, the expression of senescence markers (e.g., γH2AX, P16, P21, and P53) decreased in senescent BMSCs, while the expression of osteogenic markers, such as osteopontin (OPN) and Runx2, increased significantly, and the self-renewal ability was restored. Moreover, the QUE-loaded aging microenvironment-responsive hydrogel effectively promoted the mineralization deposition of bone plates in bone-deficient rats, increased the content of intraosseous collagen, and decreased the expression of aging-associated proteins, such as P16, γH2AX, and MMPs, while promoting the accumulation of OPN and OCN (Xing et al., 2022). However, the insufficient mechanical strength and high water dependence of hydrogels often necessitate modification to improve their applicability and stability in bone regeneration therapy. Among these, β-glycerophosphate (β-GP)-modified chitosan/collagen hydrogels are better compatible with the human body and significantly accelerate gelation in bone defects. Furthermore, this hydrogel modified by β-GP also significantly promotes the growth of periodontal stem cells (PDLSCs), reduces intracellular ROS activity, and exerts excellent antioxidant effects (Arpornmaeklong et al., 2021). Based on previous studies, Sareethammanuwat et al. (2021) doped 1%–3% β-tricalcium phosphate (β-TCP) into β-GP-chitosan/collagen hydrogels, which decreased solubility and pore size, thereby increasing the strength of ionic bonding in the β-GP-chitosan/collagen matrix, and further improving the mechanical strength of the hydrogels. Meanwhile, the addition of 1%–3% β-TCP also reduced the degradation rate and permeability, improved the pharmacokinetic properties of QUE, and made it more suitable for bone regeneration therapy. Additionally, the silk fibroin-based composite hydrogel scaffold was composed of two layers of physical silk fibroin hydrogel and one layer of a silk fibroin–calcium peroxide electrospun patch (containing 1 wt% calcium peroxide). This combination compensated for the reduction in mechanical properties resulting from the increased porosity due to QUE loading, while also improving oxygen transport within the hydrogel and exhibiting good antimicrobial properties. Notably, the composite scaffold enhanced OB viability under both normoxic and hypoxic conditions without inducing cytotoxicity (Aleemardani et al., 2023). It has been shown that QUE-embedded calcium carbonate (CaCO3) microcapsules, prepared by the co-precipitation method, can be co-prepared with β-GP and nHAP to form novel bioceramic hydrogels. The 1% CaCO3 in the material not only provided calcium ions but also stored and controlled the release of various bioactive molecules, synergizing with nHAP to reduce the porosity and degradation rate of the hydrogel. Meanwhile, 5% nHAP expanded the surface area of the pore wall, which in turn increased the mineralized nucleation sites in the hydrogel and significantly improved the compressive strength of the hydrogel. Subsequent subcutaneous implantation experiments showed that the hydrogel system had good biocompatibility in mice, did not show significant foreign body reactions, and has great potential in the clinical treatment of bone regeneration (Arpornmaeklong et al., 2023).

In recent years, nanomaterials have received significant attention due to their unique magnetic, optical, and electrical properties, with many studies focusing on the use of nanomaterials in hydrogels to address complex orthopedic diseases (Kaluđerović and Pantelić, 2023). Zhu et al. (2024) prepared QUE-loaded bioglass hydrogels with favorable radiopatterns and wide pore sizes (d ≈ 50 nm) in a water-oil biphasic layered reaction system using organic self-assembly and the sol-gel method. The incorporation of nanosized bioglass microspheres improved the biocompatibility of the material and provided an ideal surface for cell attachment and growth. This QUE-loaded bioglass hydrogel promoted the proliferation and differentiation of BMSCs, scavenged intracellular free radicals, and successfully induced osteogenic differentiation despite suppressed cyclic gene 1 expression. Additionally, it effectively promoted the repair of bone defects in periodontitis in rats, restored the height of the alveolar ridge apex, and reduced alveolar bone resorption and periodontal tissue degradation. Furthermore, the QUE-solid lipid nanoparticles prepared by the solvent emulsion diffusion method were uniformly loaded into hydrogels, and the resulting hydrogel scaffolds exhibited good bone immunomodulatory functions. The hydrogel scaffold not only possessed excellent biodegradability and *in situ* biomineralization ability but also effectively penetrated into tissues. Experimental results showed that the hydrogel scaffold with a mass fraction of 1% QUE-solid lipid nanoparticles exhibited an excellent osteogenic effect, promoting osteogenic differentiation and calcium nodule formation in BMSCs, as well as regulating macrophage polarization toward the M2 phenotype. Additionally, the hydrogel scaffold significantly promoted new bone formation, increased bone volume fraction (BV/TV) and bone mineral density (BMD), upregulated the expression of osteogenesis-related genes such as Runx2 and OCN, and suppressed OC activity in a critical-size cranial defect model rat *in vivo*, thereby regulating osteogenic-osteolytic equilibrium homeostasis toward osteogenesis (Zhou et al., 2023). In a recent study, polylactic acid-hydroxyacetic acid copolymer (PLGA) nanoparticles, prepared by the single emulsion solvent evaporation method, effectively improved the poor water solubility of QUE. Co-doping it with vancomycin into a hydrogel scaffold composed of silk fibroin protein and chitosan-thiourea slowed down the release of QUE, avoiding the potential toxicity of the initial high concentration of the drug to cells, while simultaneously reducing infection-induced bone destruction. In the MRSA-infected rat osteomyelitis model, silk fibroin protein/chitosan-thiourea-vancomycin-PLGA/QC nanoparticles significantly reduced tissue inflammation scores and increased healing scores, as evidenced by a decrease in inflammatory cells, a more organized connective tissue, and an increase in new bone formation (Jafarbeglou et al., 2024). To better mimic the biological natural bone structure, Song et al. (2025) used gelatin (Gel)-hyaluronic acid (HA) hydrogel as a matrix-loaded QUE and combined it with oriented HAP nanowires (synthesized by the solvothermal method) to form a composite hydrogel, mimicking the ordered structure of natural bone and providing topological signals to guide the directional differentiation of cells. The high strength of AHNW and the flexibility of Gel-HA make the composite hydrogel both rigid and supportive, as well as adaptable, making it highly suitable for surgical implantation. Experimental results showed that the composite hydrogel reduced the levels of senescence markers (p16, p21, γH2AX) and upregulated the expression of osteogenic genes (Col I, OCN, Runx2) in senescent BMSCs. It also elevated BV/TV and trabecular bone number (Tb.N) and maintained the integrity of fibrocartilage interface formation in an osteoporotic rat rotator cuff injury model.

It has been shown that certain metals have high bone rejuvenating properties (Yang et al., 2022). Novel MOF-modified injectable hydrogels can be obtained by doping QUE-loaded ZIF-8 metal-organic frameworks (MOFs) into temperature-sensitive hydrogels formed by dopamine-modified silk fibroin proteins with chitosan and β-GP. The release of zinc ions from the ZIF-8 framework improves the antimicrobial properties of the hydrogel, promoting osteogenesis and neovascularization, while also providing immunomodulatory capabilities. This composite hydrogel can effectively reverse the inhibition of ALP activity in PDLSCs caused by the inflammatory microenvironment, increase the expression of osteogenic genes, and enhance the synthesis and secretion of vascular endothelial growth factor (VEGF). Furthermore, when injected into the site of alveolar bone defects in rats, the repair process of the bone defects was significantly boosted, with increased expression of OPN and platelet endothelial cell adhesion molecule-1 (CD31), and more macrophages polarized toward the M2 phenotype (Yang S. et al., 2024). Additionally, some studies have used gelatin methacrylate (GelMA) hydrogels (which can be photo-cured to form a porous structure) to encapsulate ZIF-8/QUE nanoparticles, creating a structure that mimics the extracellular matrix to provide physical support for the drug and establish a slow-release microenvironment. As the inflammatory microenvironment acidifies, ZIF-8 degrades and releases QUE, which synergistically inhibits the NF-κB signaling pathway in concert with Zn^2+^ to enhance osteogenic differentiation and inhibit hyperinflammation. The hydrogel system also significantly enhanced the adhesion and migration of BMSCs, increased the expression of osteogenic differentiation markers (ALP, Runx2, OCN, alizarin-red mineralized nodules), and decreased the secretion of inflammatory factors (IL-1β and TNF-α). In in vivo experiments, ZIF-8/QUE-GelMA hydrogel elevated neonatal BV/TV and Tb.N in cranial bone defect model rats, and the resulting mature lamellar bone had a compressive strength of 12.5 MPa, which was close to the level of normal cancellous bone (Sun H. et al., 2025). In a recent study, Wang and Zhang (2025) embedded QUE within hollow spheres of titanium dioxide coated with lithium oxides on the surface (LT@Q) to synergize osteogenesis by exploiting the ionic properties (lithium ions activate the Wnt/β-catenin pathway to promote osteogenesis, and titanium ions induce the formation of bone-like apatite). LT@Q particles were co-mixed with carboxylated carbon nanotubes in Gel/chitosan hydrogels to enhance the compressive strength and electrical conductivity of the hydrogels, potentially promoting cell migration and osteogenic signaling. This multi-component synergistic targeted composite hydrogel significantly upregulated the expression of osteogenic differentiation markers (ALP, Runx2, OCN, BMP2) in OBs, while reducing the secretion of inflammatory factors (IL-1β, TNF-α).

Multiple drugs are often combined simultaneously in clinical applications to synergize bone formation. In one study, Icariin and QUE were loaded into hydrogel bilayer scaffolds prepared using the electrospinning method to regulate different aspects of bone regeneration simultaneously. This bilayer tissue-engineered scaffold addressed the deficiencies of QUE alone in cartilage repair and improved the regulation of the immune microenvironment. Experimental data showed that the scaffolds containing different concentrations of Icariin and QUE promoted the proliferation of BMSCs, resulting in effective repair of cartilage and subchondral bone. Furthermore, rats implanted with this scaffold in the osteochondral defect model of the knee joint exhibited significantly better bone volume recovery compared to the control group (Dai et al., 2024). Han’s team (Han et al., 2025) employed a similar approach, utilizing the light-curing property of GelMA to fill the inner GelMA hydrogel (loaded with QUE) into nHAP/poly (ethylene glycol-caprolactone) scaffolds (loaded with Icariin). Since GelMA degraded faster than poly (ethylene glycol-caprolactone), the constructed biphasic scaffold enabled phased drug release, creating a time-ordered synergistic effect of “QUE + Icariin.” In the early stage of *in vivo* experiments, the scaffold reduced ROS levels in OBs and regulated macrophage polarization toward the anti-inflammatory phenotype. In the later stage, QUE synergized with Icariin to significantly upregulate the expression of osteogenic genes Runx2 and OCN. Treatment of tibial defect model rats with this biphasic scaffold showed that inflammation was effectively regulated *in vivo* (with decreased levels of inflammatory factors TNF-α and IL-1β), bone repair was enhanced (increased Tb.N and decreased segregation), and the newly formed bone tissues completely filled the defect area.

**FIGURE 2:**
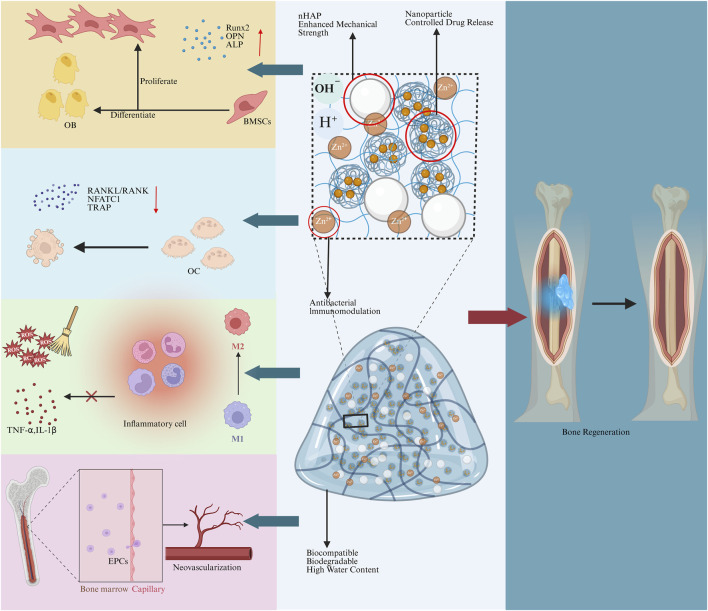
Features and Advantages of Hydrogel Delivery Systems. Hydrogels possess excellent biocompatibility, biodegradability, and high water content, making them effective drug carriers. QUE can be encapsulated in nanoparticles to achieve controlled drug release, which is then further loaded into the hydrogel. Incorporating nHAP into the hydrogel enhances its mechanical strength, while the addition of metal ions improves its antibacterial properties and confers immunomodulatory functions. Modifications to the hydrogel can also impart pH responsiveness and other characteristics. The hydrogel designed with composite materials can enhance the proliferative and differentiative effects of QUE on BMSCs, promoting the expression of osteogenic-related proteins such as Runx2, OPN, and ALP. Additionally, it can further enhance the inhibitory effects of QUE on OC cell activity and function, as well as improve QUE’s anti-inflammatory, antioxidant stress, and pro-angiogenic effects. After loading QUE, the composite material-designed hydrogel exhibits significant therapeutic effects on bone diseases. Abbreviations: BMSCs: Bone Marrow Mesenchymal Stem Cells; OC: Osteoclast; OB: Osteoblast; ALP: Alkaline Phosphatase; TRAP: Tartrate-Resistant Acid Phosphatase; ROS: Reactive Oxygen Species; RANKL: Nuclear Factor κB Receptor-Activated Ligand; RANK: Receptor Activator of Nuclear Factor - κB Receptor; Runx2: Runt-Related Transcription Factor 2; OPN: Osteopontin; NFATc1: Nuclear Factor of Activated T Cells Cytoplasmic 1; EPCs: Endothelial Progenitor Cells; nHAP: Nanohydroxyapatite; TNF-α: Tumor Necrosis Factor-α; IL-1β: Interleukin-1β.

With the continuous optimization and improvement of hydrogel materials, their potential for combined application with QUE in bone regeneration and tissue repair has been significantly expanded. These materials can not only enhance the role of QUE in regulating the immune microenvironment but also improve its ability to promote osteogenesis and inhibit bone resorption. Given the wide range of applications and functions of hydrogel materials in QUE delivery systems, we have included a schematic diagram (Figure 2) to systematically demonstrate their multiple advantages. For a clear and comprehensive comparison of these hydrogel systems, their key features are summarized in Table 1. In recent years, the application value of hydrogel-loaded QUE has become increasingly evident, and it is expected to provide strong support for innovation and development in the field of bone regeneration in the future, further promoting the popularization and optimization of clinical treatments.

**TABLE 1:** Overview of quercetin-loaded hydrogel systems for bone regeneration.

Types of drug delivery systems	Primary materials/Trim	Preparation method	Highlighting advantages	Drugs for loading	Main biological effects	References
Hydrogel	FA modified QUE microemulsion-thermo hydrogel	Microemulsion preparation + cold solution method	Thermal responsiveness; excellent targeting; good adaptability	QUE	Reduced collagen degradation and OC activity; reestablished helper T cell 17/regulatory T cell homeostasis; achieved precise repair of periodontal bone defects	Li J. et al. (2024)
	MMP-responsive microenvironmental targeting hydrogels for aging	Thermally induced self-assembly	MMP responsiveness	QUE	Decreased senescence markers (γH2AX, P16, etc.) and upregulated osteogenic markers (Runx2, OPN); promoted bone plate mineralization and increased collagen content	Xing et al. (2022)
	β-GP modified chitosan/collagen hydrogel with β-TCP	Thermally induced sol-gel transformation method	Rapid gelation; excellent mechanical strength; low degradation rate	QUE	Promoted the growth of hPDLSCs and reduced intracellular ROS; optimized QUE pharmacokinetics and enhanced bone regeneration applicability	Arpornmaeklong et al. (2021), Sareethammanuwat et al. (2021)
	Silk protein composite hydrogel (with calcium peroxide electrospun patch)	Ultrasound-induced physical gelation + electrostatic spinning method	Good oxygen transport; excellent mechanical properties; excellent antimicrobial properties	QUE	Enhanced OB viability; was non-cytotoxic; adapted to the microenvironment of hypoxic bone defects	Aleemardani et al. (2023)
	Bioceramic hydrogel (QUE-CaCO3+β-GP+nHA)	Thermally induced sol-gel transformation method	Release of Ca2+, bioactive molecules; improve compressive strength of large	QUE	Exhibited excellent biocompatibility (no foreign body reaction) and was suitable for bone regeneration	Arpornmaeklong et al. (2023)
	Bio glass injectable hydrogel	Sol-gel method + light-curing cross-linking method	Wide pore size (≈50 nm); radiolucent morphology; excellent biocompatibility; provides surface for cell attachment	QUE	Promoted the proliferation and differentiation of BMSCs and scavenged free radicals; restored alveolar ridge height and reduced bone resorption	Zhu et al. (2024)
	Solid lipid nanoparticle composite hydrogel scaffolds	Solvent emulsification diffusion + temperature-induced sol-gel transformation	Excellent biodegradability; high in situ mineralization; immunomodulation	QUE	Promoted osteogenic differentiation and calcium nodule formation in BMSCs; increased BV/TV and BMD, upregulated Runx2/OCN, and inhibited OC activity	Zhou et al. (2023)
​	Silk protein/chitosan thiourea hydrogel scaffolds (loaded with PLGA nanoparticles)	Emulsification-solvent evaporation method + freeze-drying method	Excellent water solubility; dual-drug slow release (antimicrobial + osteogenic); adapted to MRSA-infected environments	QUE + vancomycin	Reduced tissue inflammation scores and improved healing scores; reduced inflammation levels; promoted new bone formation	Jafarbeglou et al. (2024)
	Gelatin-hyaluronic acid/oriented HAP nanowire composite hydrogel	Solvent heat method + crosslinking curing method	Modeling natural bone structure; providing topological signals; rigid-flexible bonding	QUE	Reduced markers of senescent BMSCs and upregulated osteogenic genes; elevated BV/TV and Tb.N and maintained fibrocartilage interface integrity	Song et al. (2025)
	ZIF-8 modified temperature-sensitive hydrogel (dopamine silk protein + chitosan)	Heat-induced sol-gel conversion + cross-linking curing method	Zn2+ orchestrates antimicrobial, osteogenic, angiogenic, and immunomodulatory	QUE	Reversed inflammatory suppression of PDLSCs; upregulated osteogenic genes and VEGF; increased OPN/CD31 expression, promoting bone defect repair	Yang S. et al. (2024)
	ZIF-8/QUE/GelMA hydrogel	One-pot method + light-curing cross-linking method	Simulated extracellular matrix; inflammatory acidification responsiveness; Zn2+ synergy	QUE	Enhanced BMSCs adhesion migration and elevated ALP/Runx2/OCN; achieved a compressive strength of new bone up to 12.5 MPa (close to normal cancellous bone)	Sun Y. et al. (2025)
	Gelatin/chitosan hydrogel (LT@Q + carboxylated carbon nanotubes)	Chemical oxidation + cross-linking curing method	Li+, Ti+ synergistically promote osteogenesis; excellent compressive strength and electrical conductivity	QUE	Upregulated ALP/Runx2/OCN/BMP2 and reduced IL-1β/TNF-α	Wang and Zhang (2025)
	Bilayer/Biphasic hydrogel stents	Fused deposition molding 3D printing method + light-curing cross-linking method	QUE, icariin phased release; synergistic regulation of bone and cartilage repair	QUE + icarin	Promoted the proliferation of BMSCs to repair cartilage and subchondral bone; reduced the level of inflammatory factors; increased Tb.N to fill the defective area	Dai et al. (2024), Han et al. (2025)

### 3.2 Hydroxyapatite

HAP is a calcium phosphate mineral containing hydroxyl ions, with the chemical formula Ca_10_(PO_4_)_6_(OH)_2_. Its chemical composition and crystal structure are highly similar to those of the main inorganic constituents of the human skeleton and teeth, making it an important member of the apatite family of minerals. HAP combines with organic substrates (mainly collagen) in the form of nanoscale crystals to form the basic structure of bone, accounting for more than 50% of the total weight of bone (Von et al., 2019; Ulian et al., 2021). Due to these properties, HAP materials have excellent compatibility in living organisms, with a very low risk of immune rejection and inflammatory reactions (Kumar and Mohanty, 2022). Based on this, the use of HAP as a scaffold material can provide a favorable microenvironment for osteoblast attachment and proliferation, further enhancing new bone generation and the repair of defects (Alkaron et al., 2024). Additionally, HAP has a unique chemical structure, rich surface functional groups, good bioactivity, and a porous nanoscale structure, which can be combined with other materials in various ways to produce composites with excellent performance. HAP and its composites have been widely used to improve the stability, solubility, and cellular affinity of drugs, significantly enhancing QUE treatment for bone regeneration (Kataoka et al., 2024).

It was found that HAP microbeads developed by electrostatic spraying could enhance the ALP activity of MG-63 cells, as well as the expression levels of osteogenic marker genes such as ALP and Runx2, after loading QUE. Implantation of QUE-loaded HAP microbeads into cranial bone defect model rats significantly increased the percentage of bone growth area, BV/TV, and BMD, as well as expanded the OCN-positive area (Lee et al., 2023). However, HAP obtained by traditional synthetic methods often suffers from shortcomings such as insufficient purity and high crystallinity, which greatly affect the adsorption properties and bioactivity of the material. It is worth noting that HAP prepared by the montmorillonite phase transition method not only effectively overcomes these drawbacks, but also maintains the good radical scavenging activity of QUE. Experiments have shown that QUE-loaded HAP can significantly enhance the differentiation and activity of OBs, upregulate the expression of osteogenic markers such as ALP, Col I, OCN, and OPN, and inhibit the proliferation of OC precursors. Additionally, it reduces the expression levels of CTSK, pro-inflammatory cytokine IL-6, and transforming growth factor β1 in OCs, exerting anti-inflammatory effects and inhibiting OC differentiation (Forte et al., 2016). Furthermore, alginate solution as a matrix can enhance the cross-linking ability of HAP nanoparticles, enabling QUE-containing HAP microspheres to more effectively promote Ca^2+^ accumulation and improve the expression of osteogenic markers such as Col I, ALP, and OCN, along with their corresponding genes (Kim et al., 2022). Building on this, by combining the gelatinous alginate shells with calcium-deficient hydroxyapatite (CDHA,a highly osteoconductive material with a calcium-to-phosphorus ratio lower than the stoichiometric ratio) prepared by a self-curing reaction and generating core-shell microbeads using three-dimensional printing technology, a multifunctional CDHA-alginate core-shell system can be obtained. This system is not only capable of achieving simultaneous drug-cell delivery but also ensures long-term QUE release while maintaining cell viability (Raja et al., 2021).

HAP alone has certain mechanical property limitations, and combining HAP with certain biomaterials can develop composites with improved properties (Mashak et al., 2022). For instance, Song et al. (2020) prepared porous sponges composed of QUE, duck paw collagen, and HAP by lyophilization. QUE/duck paw collagen/HAP exhibited ideal porosity (>70%) and significantly promoted the proliferation of BMSCs at a concentration of 25 μM QUE. It also upregulated the expression of bone-specific genes such as OCN, Col I, and Runx2, showing enhanced cell proliferation and osteogenic differentiation. Further implantation of the sponge into rats with cranial bone defects improved the bone microenvironment, increased new calcified bone formation, and enhanced bone density and volume. Additionally, α-calcium sulfate hemihydrate/nHAP is well-suited for bone tissue engineering, as it not only possesses good osteoconductivity and inducibility to promote bone regeneration and repair but also enhances the osteogenic effect of QUE. *In vitro* assessment of bone regeneration effects showed that QUE-loaded α-calcium sulfate hemihydrate/nHAP significantly promoted the differentiation of OBs and BMSCs, increased the number of Runx2-positive cells, and raised serum calcium concentration (Ren et al., 2022). Moreover, decellularized goat lung scaffolds were crosslinked and modified with QUE and nHAP, and the final QUE-crosslinked, nHAP-modified decellularized scaffolds integrated effectively with human tissues. This novel scaffold reduced immune rejection and cytotoxicity, inhibited the differentiation of OC progenitor cells and the activity of mature OCs, and enhanced cell adhesion, proliferation, and differentiation of BMSCs, as well as increased ALP activity and calcium deposition levels (Gupta et al., 2017). It has been shown that Gel/yellow yarrow improves the high brittleness of high-content nHAP scaffolds and enhances the bone regeneration effect of QUE treatment through synergistic effects. In further experiments, QUE-loaded Gel/yellow yarrow gel/nHAP scaffolds significantly increased cell viability and ALP activity in BMSCs, and also upregulated the expression of osteogenesis-related genes such as basic fibroblast growth factor, Osterix, secreted phosphoprotein 1, osteocalcin, Col I, and Runx2 (Madani et al., 2023). In addition, Kiakojoori et al. (2022) utilized polyether ether ketone (PEEK)/PLGA copolymer particles to encapsulate QUE, thus achieving controlled drug release. Meanwhile, their incorporation into calcium phosphate bone cement with good bioactivity synergistically improved the mechanical properties of PEEK/PLGA particles and provided stable support for filling bone defect sites. In the subsequent *in vitro* osteogenic assay, the calcium phosphate bone cement loaded with QUE-PEEK/PLGA particles significantly enhanced the proliferation and activity of human dental pulp stem cells, and upregulated the expression of osteogenic genes such as OCN, OPN, and ALP. By functionalizing HAP, its properties can be optimized, which in turn improves its applicability in bone repair. For example, the addition of silk protein to QUE-loaded nHAP scaffolds can enhance the biocompatibility and biodegradability of the scaffolds. This QUE-loaded nHAP/silk protein scaffold not only promotes the proliferation and differentiation of BMSCs but also enhances intracellular ALP activity and the expression of osteogenesis-related genes such as Col I, OCN, and Runx2. Bone mineralization activity and bone matrix formation were significantly enhanced in rats with cranial bone defects after treatment with this scaffold implantation (Song et al., 2018). Additionally, nHAP can be functionalized with alendronate, an anti-osteoporotic drug, to alleviate the H2O2-induced decrease in OB activity. nHAP, loaded with alendronate and QUE, inhibits OC differentiation and the expression of inflammatory mediators such as TNF-α and ROS, while up-regulating the expression of OCN and Runx2. This functionalization has good antioxidant properties and contributes to osteoinductive effects (Forte et al., 2017).

In conclusion, the combination of HAP and QUE has attracted widespread attention in the treatment of bone metabolic diseases, mainly because they exhibit significant synergistic effects in terms of biocompatibility, bioactivity, drug delivery systems, and multifunctional composite material development. This combination not only effectively promotes bone regeneration and inhibits bone resorption but also enables the sustained action of drugs through a slow-release mechanism, addressing the clinical demand for efficient and safe therapeutic materials. For a concise and systematic overview of these systems, their key characteristics are summarized in Table 2. As research progresses, HAP and QUE composites are expected to play an increasingly critical role in the treatment of bone metabolic diseases.

**TABLE 2:** Hydroxyapatite-based systems for quercetin delivery in bone repair.

Types of drug delivery systems	Primary materials/Trim	Preparation method	Highlighting advantages	Drugs for loading	Main biological effects	References
Hydroxyapatite	HAP microbeads	Electrostatic spraying	Mimics inorganic components of bone for excellent biocompatibility	QUE	Elevated ALP activity and Runx2 expression in MG-63 cells; increased bone growth area, BV/TV and BMD, and expanded OCN-positive area	Lee et al. (2023)
	HAP	Direct precipitation + phase transition transformation method	High purity; moderate crystallinity; free radical scavenging activity; good adsorption properties; improved QUE bioactivity	QUE	Promoted OB differentiation, upregulated ALP, Col I, OCN, OPN; inhibited OCP proliferation and CATK, IL-6, TGF-β1 expression in OC	Forte et al. (2016)
	CDHA-alginate core-shell beads	Self-curing reaction + 3D printing	Drug-cell co-delivery; QUE long-lasting release	QUE	Promoted Ca2+ accumulation and up-regulated COL-1, ALP, OCN; ensured cell viability and adapted to the long-lasting needs of bone defect repair	Kim et al. (2022), Raja et al. (2021)
	Duck paw Collagen/HAP porous sponge	Lyophilization	High porosity (>70%); excellent biocompatibility	QUE	Increased neo-calcified bone formation and enhanced bone density and bone volume	Song et al. (2020)
	α-Calcium sulfate hemihydrate/n-HAP complexes	Molding and curing method	Osteoconductive, inducible	QUE	Promoted differentiation of OB and BMSCs, increased the number of RUNX2-positive cells, and raised serum calcium concentration	Ren et al. (2022)
	QUE cross-linked, nHAP-modified decellularized goat lung scaffolds	Decellularization + cross-linking modification	Excellent human tissue integration; low incidence of immune rejection; low cytotoxicity	QUE	Inhibited OC progenitor cell differentiation and OC activity; enhanced BMMSCs adhesion, proliferation and differentiation; increased ALP activity and calcium deposition	Gupta et al. (2017)
	Gelatin/Yellow yarrow Gum/nHAP scaffolding	Freeze-drying method	Moderate brittleness; excellent mechanical properties; excellent bioactivity	QUE	Increased BMSCs viability and ALP activity; up-regulated osteogenic genes such as bFGF, SP7, SPP1, and BGLAP	Madani et al. (2023)
	PEEK/PLGA particulate-calcium phosphate cement composite scaffolds	Solvent evaporation method + hybrid curing method	Mechanical support stabilization	QUE	Enhanced DPSCs proliferation and activity, upregulated OCN, OPN, ALP expression; provided stable support for bone defects	Kiakojoori et al. (2022)
	nHAP/Silk protein scaffold	Freeze-drying method	Excellent biocompatibility; high osteoinductivity	QUE	Promoted the proliferation and differentiation of BMSCs, increased ALP activity and COL1, OCN, RUNX2 expression; enhanced bone mineralization and matrix formation	Song et al. (2018)
	Alendronate-functionalized HAP nanocrystals	Coprecipitation	Improvement of OB hypoactivity; synergistic antioxidant and osteogenesis	QUE	Inhibited OC differentiation and expression of lactate dehydrogenase, TNFα, ROS; upregulated OCN, RUNX2, demonstrating both anti-osteoporosis and pro-osteogenic effects	Forte et al. (2017)

### 3.3 Biofunctional scaffolds

Biofunctional scaffolds are engineered frameworks that combine biological activity and specific functions to support tissue repair or drug delivery, typically made from natural or synthetic biopolymers. These scaffolds possess a three-dimensional porous structure that mimics the extracellular matrix *in vivo*, providing an ideal microenvironment for cell attachment, proliferation, migration, and differentiation (Rifai et al., 2023; Zhu et al., 2023). Due to these properties, biofunctional scaffolds have been widely used in bone tissue engineering and regenerative medicine. They serve as three-dimensional support structures and growth platforms for cells to aid in the regeneration and repair of bone, cartilage, skin, and other tissues. Additionally, they actively participate in and promote tissue repair through various mechanisms, such as releasing growth factors, regulating immune responses, promoting angiogenesis, guiding cell differentiation, and improving the microenvironment (Liu et al., 2023; Cota Quintero et al., 2025). Furthermore, the material composite design of biofunctional scaffolds, with the aid of 3D printing technology, allows precise regulation of the scaffolds’ mechanical properties and enhances their bonding with host tissues, enabling personalized design to meet patient-specific needs (Liu H. et al., 2024). Therefore, biofunctional scaffolds have become indispensable materials in the field of bone repair, especially in large-area bone defects, poor fracture healing, and bone grafting, playing a key role.

Titanium and titanium alloys are the primary materials used for orthopedic implants; however, the bioinertness of titanium alloys limits their effectiveness in long-term implantation. As a result, many researchers have focused on developing titanium implants that meet clinical needs. For example, Wang et al. (Wang et al., 2021b) prepared a QUE-loaded polyelectrolyte multilayer-coated titanium scaffold using the layer-by-layer self-assembly technique. The surface of this scaffold features polyethyleneimine as the base layer, which inhibits bacterial growth and reduces the risk of infection. Additionally, it has a strong binding force that allows chitosan, HA, and QUE coatings to adhere to the titanium surface, forming a multilayered bioactive membrane. This membrane improves the hydrophilicity and flexibility of the scaffold surface while enabling the sustained controlled release of QUE. This QUE-loaded polyelectrolyte multilayer-coated titanium scaffold effectively promoted the adhesion and proliferation of MC3T3-E1 cells, enhanced ALP activity, increased the expression of osteogenic proteins such as Col I, Runx2, and OCN, and improved the mineralization level of the extracellular matrix. Ovariectomized rats treated with QUE-loaded polyelectrolyte multilayer-coated titanium scaffolds showed a significant increase in peripheral bone volume, along with abundant Tb and osteoid regeneration, as well as a much higher percentage of bone formation area. In another study (Liu et al., 2022), surface modification of 3D-printed Ti6Al4V by alkaline-thermal and hydrothermal treatments led to the preparation of a novel titanium alloy scaffold. The nanostructures of this scaffold not only provide sufficient binding sites to immobilize QUE but also adsorb specific proteins, which in turn create a more favorable environment for cell growth. The experimental results showed that the QUE-loaded Ti6Al4V scaffolds were suitable for BMSC adhesion, could regulate macrophage polarization toward the M2 phenotype, promoted osteogenic differentiation of BMSCs, and significantly increased the volume of new bone formation in bone defect model rats, while decreasing the level of inflammation. Additionally, the adhesive and multifunctional groups of the polydopamine (PDA) coating can firmly anchor silver nanoparticles and QUE onto the porous titanium surface, preventing free ion toxicity. The resulting coating activated osteogenic signaling pathways such as Wnt/β-catenin and inhibited the differentiation of bone marrow-derived macrophages to OC, while promoting endothelial cell migration and lumen formation. It significantly promoted osteogenesis and inhibited inflammation after implantation into MRSA-infected femur model rats, demonstrating excellent bone-implant interface stability (Yang et al., 2025). In the latest study, Hu’s team (Hu et al., 2023) first loaded an appropriate concentration of QUE into a chitosan/Gel composite membrane to improve the poor water solubility of the drug, then coated it on the surface of titanium wafers by electrophoretic deposition. The obtained scaffold showed a strong bond between the membrane layer and titanium substrate, as well as a slow-release function (initial burst release + sustained release), suitable for the long-term antimicrobial needs of the implant. The experimental results showed that this scaffold had a significant regulatory effect on BMSCs, increasing their activity and reducing apoptosis, while inducing apoptosis of MG-63 cells in a dose-dependent manner.

In addition, traditional guided tissue regeneration membranes (GTR membranes) have the disadvantages of mismatched degradation rates, insufficient mechanical properties, and limited ability to promote tissue regeneration. To address these issues, Hu et al. (2024) used an electrospinning method to prepare a fiber membrane composite scaffold from polycaprolactone (PCL) mixed with QUE. The scaffold was non-toxic to cells and promoted osteogenic differentiation, migration, and calcium deposition in PDLSCs, as well as enhanced ALP activity and the expression of osteogenic marker genes such as ALP, OPN, and OCN. Meanwhile, its degradation rate was moderate, and it possessed good mechanical properties, such as ultimate tensile strength and Young’s modulus, which allowed it to maintain its shape and structure in body tissues for a long time, providing a suitable and stable spatial environment for bone tissue regeneration. Additionally, the rate of new bone formation, BV/TV, and BMD levels were significantly improved after implantation of this composite scaffold in rats with periodontal bone defects. In another study (Toulou et al., 2024), the use of TCP and KCl further enhanced the scaffold properties. This method improved the mechanical strength of the scaffolds and increased the open porosity, roughness, and free surface area, thus promoting cell adhesion. Furthermore, TCP and KCl also regulated the degradation rate of the scaffold to prevent the burst release of drugs. This modified scaffold loaded with QUE demonstrated excellent osteogenic effects, enhanced cell viability and adhesion of OBs, and inhibited the proliferation and cell viability of MG-63 cells. In addition, mesoporous calcium silicate nanoparticles and calcium sulfate were used to regulate porosity and pore size, enhance the stability of QUE in biological tissues, and improve the stability of scaffolds. Meanwhile, mesoporous calcium silicate nanoparticles can release Si ions, which activate the expression of bone-related proteins. Incorporating mesoporous calcium silicate nanoparticles and calcium sulfate into the QUE-PCL scaffold improved its biocompatibility. In in vitro experiments, the scaffold not only enhanced the proliferation and adhesion of Wharton’s gum MSCs but also significantly elevated the level of calcium deposition (Huang et al., 2021).

To overcome the limitations of conventional drugs, such as bisphosphonates, in the treatment of osteoporosis, researchers have developed QUE-loaded CDHA scaffolds as an alternative to topical drug administration to reduce the toxic side effects of drugs. The scaffold is made of α-TCP paste doped with QUE and has good mechanical properties, with a compressive strength similar to that of human cancellous bone, reaching 20 ± 1.8 MPa. Additionally, the scaffold has a porosity suitable for human tissues (58.6 ± 1)%, which provides an advantageous spatial environment for cell growth, proliferation, and nutrient exchange. The osteogenic effect is particularly excellent when loaded with 200 μM QUE. In an *in vitro* biological evaluation, QUE-loaded CDHA scaffolds significantly enhanced OB proliferation and mineralization, up-regulated the expression of key osteogenic genes such as Runx2, Col I, and ALP, while inhibiting OC proliferation and TRAP activity (Tripathi et al., 2015). Furthermore, an increasing number of biomaterials are being used in the fabrication of biofunctional scaffolds to help alleviate problems such as immune rejection, secondary surgery, and infection. Among them, the poly-L-propylene glycol scaffold printed by a fused deposition modeling system has a square pore mesh structure, which can form a poly-L-propylene glycol/PDA/QUE composite scaffold after functionalizing the PDA layer and loading QUE. This scaffold combines the advantages of synthetic polymers and biomaterials, not only having good compression properties that mimic the mechanical environment *in vivo* to safeguard normal cellular activities, but also facilitating the attachment and growth of MC3T3-E1 cells. It enhances their ALP activity and calcium deposition, while simultaneously increasing the expression of osteogenesis-related genes and proteins such as Runx2, ALP, Col I, and OCN (Chen et al., 2019). QUE-coated cockroach wing scaffolds have attracted much attention in recent studies. Due to the good biocompatibility and biodegradability of cockroach wings and the appropriate pore spacing (average pore spacing of 16 μm) of the ridge pattern on their surfaces, they are advantageous for both drug loading and cell adhesion. After plasma and QUE treatments, it was observed that this QUE-coated cockroach wing scaffold was able to enhance cell viability and ALP activity of BMSCs, increase the expression of key osteogenic genes such as Col I, BMP2, Runx2, secreted phosphoprotein 1, osteocalcin, and significantly increase the calcium content in OBs (Mostofi et al., 2024).

As a supportive tool, biofunctional scaffolds provide physical support for cells and enhance the bioavailability of QUE. Future research should focus on optimizing the scaffold’s architecture and material composition to improve the stability and release kinetics of QUE, as well as exploring its therapeutic potential in complex pathological environments. To facilitate a clear comparison of these platforms, their core features are outlined in Table 3. Leveraging synergistic interactions with other bioactive molecules, QUE-loaded biofunctional scaffolds hold promise as a significant breakthrough in the field of bone tissue engineering.

**TABLE 3:** Biofunctional scaffolds for quercetin delivery in bone engineering.

Types of drug delivery systems	Primary materials/Trim	Preparation method	Highlighting advantages	Drugs for loading	Main biological effects	References
Biofunctional scaffolds	Polyelectrolyte multilayer coated titanium scaffold (polyethyleneimine base layer)	Layer-layer self-assembly technology	Strong bacterial inhibition; sustained controlled release QUE; excellent hydrophilicity; excellent flexibility	QUE	Promoted cell adhesion and proliferation; enhanced ALP activity and ColI, RUNX2, and OCN expression; increased peripheral bone volume and Tb regeneration	Wang et al. (2021b)
	Nanostructured Ti6Al4V scaffolds	3D printing + alkaline heat treatment - hydrothermal method	Fixed QUE; optimized cell growth environment	QUE	Regulated macrophage polarization to M2; promoted osteogenic differentiation of BMSCs; increased the volume of new bone formation; decreased inflammation levels	Liu et al. (2022)
	Polydopamine-coated porous titanium scaffolds	Alkali thermal treatment + silver nanoparticle reduction method	Antimicrobial-osteogenic-vascularizing triple function	QUE	Activated the Wnt/β-catenin pathway; enhanced bone-implant interface stability; inhibited drug-resistant bacterial infection	Yang et al. (2025)
	Chitosan/gelatin composite film coated titanium sheets	Electrophoretic deposition	“Initial burst + sustained release” for long-term antimicrobial needs	QUE	Increased BMSCs activity and reduced apoptosis; induced MG-63 apoptosis in a dose-dependent manner	Hu et al. (2023)
	QUE-PCL electrospun fiber membrane support	Electrospinning	Moderate degradation rate; excellent mechanical properties (ultimate tensile strength, Young’s modulus); provides space for bone regeneration stabilization	QUE	Promoted hPDLSCs osteogenic differentiation, migration and calcium deposition; elevated ALP, OPN, OCN expression; increased new bone formation, BV/TV, and BMD	Hu et al. (2024)
	TCP/KCl enhanced QUE-PCL holder	Melt Blending-3D printing method	Excellent mechanical strength; high open porosity; moderate degradation rate	QUE	Enhanced OB viability and adhesion; inhibited osteosarcoma cell proliferation	Toulou et al. (2024)
	Mesoporous calcium silicate/calcium sulfate-PCL scaffolds	Melt Blending-3D printing method	Activation of bone-associated proteins by Si ions; moderate porosity and pore size; excellent QUE stability and slow-release properties	QUE	Enhanced the proliferation, adhesion and calcium deposition of Wharton’s jelly mesenchymal stem cells	Huang et al. (2021)
	Poly (L-propylene glycolide)/PDA composite stent	Fused deposition molding 3D printing method + surface modification method	Square pore mesh structure; excellent compressive properties; simulates in vivo mechanical environment	QUE	Promoted cell proliferation and attachment; enhanced ALP activity and calcium deposition; up-regulated Runx-2, col I, OCN and other genes and protein expression	Chen et al. (2019)
	CDHA scaffolds	3D printing + cement curing method	High compressive strength; suitable for cell growth, nutrient exchange	QUE	Upregulated osteogenic genes such as RUNX-2 and col I; inhibited OC proliferation and TRAP activity	Tripathi et al. (2015)
	QUE coated cockroach wing holder	Plasma treatment + solution immersion method	Excellent biocompatibility and degradability; suitable for cell adhesion	QUE	Enhanced BMSCs viability and ALP activity; up-regulated osteogenic genes such as col I, BMP2, Runx2; increased OB calcium content	Mostofi et al. (2024)

### 3.4 Phospholipid-based carriers

Phospholipid-based carriers are a class of drug delivery systems constructed using phospholipid materials. Their function is based on the amphiphilic property of phospholipids, and after combining with other substances through self-assembly, graft copolymerization, Michael addition, and other reactions, they can form drug delivery carriers with specific structures and functions (Kuperkar et al., 2022). Phospholipid-based carriers can not only encapsulate water-soluble substances but also embed fat-soluble substances in phospholipid bilayer membranes, thereby achieving efficient encapsulation of ingredients (Mehta et al., 2023). In addition, phospholipid-based carriers exhibit excellent biocompatibility and controlled release capabilities, ensuring the stability and absorption efficiency of active ingredients (Gbian and Omri, 2022). Based on these characteristics, phospholipid-based carriers are not only suitable for encapsulating and delivering active ingredients such as drugs, growth factors, genes, and plant extracts but also enable the precise release of drugs, making them one of the key technologies in modern medicine and bone tissue engineering (Filipczak et al., 2021). With excellent stability and efficient delivery capabilities, the combination of phospholipid-based carriers and QUE can effectively overcome the limitations of QUE in promoting bone regeneration and provide a more effective strategy for bone regeneration therapy.

In recent years, techniques have emerged to improve QUE bioavailability using phospholipid-based carriers. Phospholipid complex nanoparticles were prepared by Abd El-Fattah et al. (2017) using the thin-film hydration method. The particles not only significantly improved the solubility of QUE but also enhanced its stability in the organism by efficiently encapsulating QUE (encapsulation rate of 98.4%). These particles were able to interact with the phospholipid bilayer of the cell membrane, facilitating the delivery of QUE into the cell. When loaded with a high dose of free QUE (50 mg/kg), the phospholipid complex nanoparticles significantly increased serum calcium and phosphorus levels in de-ovulated rats, resulting in improved bone metabolic status. Similarly, Pandit et al. (2020) prepared phospholipid transfersomal vesicles encapsulating QUE using the thin-film hydration method. With excellent elasticity and deformability, these transfersomes were able to easily penetrate the skin barrier, enabling transdermal absorption of the contained drug. Based on this, the uniform distribution and immobilization of the delivery bodies in chitosan membranes provided physical protection for drug delivery and storage, and also promoted drug release. The experimental results showed that this QUE-containing deliverer-chitosan membrane significantly reduced OB apoptosis, elevated serum ALP levels, and effectively restored serum calcium and phosphorus concentrations in osteoporosis model rats. Meanwhile, significant enhancements in bone density, thickness, weight, and bone tensile strength were observed in the rats.

Studies have shown that FA receptors are often highly expressed on the surface of tumor cells, allowing for the development of highly targeted anti-osteosarcoma drugs through FA modification of QUE-loaded liposomes (Zwicke et al., 2012). It is worth noting that FA-QUE liposomes not only inhibit the proliferation, migration, and invasion of osteosarcoma cells but also promote their apoptosis. In tumor model nude mice, these liposomes effectively inhibited tumor growth (Jing et al., 2022). Additionally, Xing et al. (2023) prepared a liposome with targeting properties using 1,2-distearoyl-sn-glycero-3-phosphate ethanolamine, polyethylene glycol 2000, and egg yolk lecithin as the basic structural framework. A high affinity for bone tissue was achieved by linking it with (DSS)_6_, which has excellent biocompatibility and drug-carrying properties and can maintain high stability in complex *in vivo* environments. This functionally modified liposome, loaded with QUE, excelled in promoting bone regeneration in senescent mice, not only eliminating senescent cells in bone tissue but also increasing the rate of bone formation and enhancing the expression of osteogenic markers such as OCN and Runx2. It also significantly increased Tb volume in senescent mice and boosted the number of calcified nodules formed by BMSCs. In a recent study (Zhang C. et al., 2025), researchers created a microemulsion system of QUE with glyceryl tributyrate, polyethylene glycol 15 hydroxystearate, and polyethylene glycol 400 by aqueous titration to optimize the water solubility and uptake efficiency of QUE. This was then complexed with caffeic acid phenethyl ester liposomes to co-exploit the pro-bone regeneration effect. It was further co-loaded with silver nanoparticles in a composite hydrogel formed by chitosan, Porosam 407, and poly-N-isopropylacrylamide through physical cross-linking. This utilized the temperature/pH-sensitive properties of the hydrogel to achieve localized targeted delivery and long-lasting retention in tissues. This composite phospholipid system preferentially releases QUE for anti-inflammation in an acidic environment and slowly releases caffeic acid phenethyl ester to assist osteogenesis in a neutral environment, thus avoiding insufficient anti-inflammation in the early stage and delayed osteogenesis in the late stage. *In vitro* results showed that the complex phospholipid system reduced the proportion of pro-inflammatory phenotypic macrophages and ROS levels, while increasing ALP activity and OCN gene expression in PDLSCs. In the subsequent *in vitro* assessment of osteogenic effects, periodontitis model rats showed a significant reduction in inflammatory cell infiltration and restoration of collagen fiber alignment, accompanied by improved alveolar bone BV/TV and expansion of the OPN-positive area.

In addition, phospholipid-based carriers can be used in combination with bone tissue-engineered scaffolds to improve the therapeutic efficacy of QUE. In a recent study (Bose et al., 2024), researchers used solid lipid nanoparticles to encapsulate QUE, which enabled controlled release of the drug. β-TCP 3D scaffolds were also employed to enhance the precision of drug action and reduce toxic side effects on normal tissues. Furthermore, the β-TCP scaffold provides proper mechanical support to the bone defect site and guides the growth of bone tissue. This QUE-loaded multifunctional phospholipid-scaffold composite system exhibits selective cytotoxicity when used in conjunction with vitamin D3, which promotes OB proliferation and differentiation while decreasing osteosarcoma cell activity, reducing the expression of TRAP in OCs, and inhibiting the formation of resorption pits. In further studies, Nermeen (Kamal et al., 2023) combined a nanolipid-structured carrier with a calcium phosphate scaffold and modified QUE with phospholipids using soybean phosphatidylcholine. The drug delivery system he designed showed good histocompatibility and overall improved the solubility, stability, and biophilicity of QUE. In subsequent *in vivo* implantation experiments, this nanolipid carrier-calcium phosphate scaffold based on phospholipid-modified QUE significantly promoted the healing of femoral defects in rats, enhanced the formation of bone calluses, and effectively reduced the production of abnormal fibrous tissue.

Phospholipid-based carriers have excellent biocompatibility and can effectively protect QUE from degradation, enhance its solubility, and facilitate absorption and targeted delivery. By combining the advantages of phospholipid-based carriers, the stability and bioavailability of QUE *in vivo* have been significantly improved, allowing it to be more precisely applied to the target site. For a comprehensive and structured summary, the critical attributes of these systems are listed in Table 4. The application of phospholipid-based carrier-loaded QUE in bone regeneration is promising, and future research could focus on optimizing carrier design to achieve more precise targeted delivery, as well as evaluating its long-term efficacy and safety in the treatment of complex bone injuries.

**TABLE 4:** Phospholipid-based carriers for quercetin delivery in bone repair.

Types of drug delivery systems	Primary materials/Trim	Preparation method	Highlighting advantages	Drugs for loading	Main biological effects	References
Phospholipid-based carriers	Phospholipid complex nanoparticles	Membrane hydration method	High encapsulation rate (98.4%); suitable for cellular uptake; high QUE stability	QUE	Increased serum calcium and phosphorus levels; improved bone metabolic status	Abd El-Fattah et al. (2017)
	Phospholipid vesicle transporter-chitosan membrane complexes	Membrane hydration method	Highly penetrating; combines physical protection and drug release; combines transdermal delivery with membrane carriers	QUE	Reduced OB apoptosis; elevated serum ALP levels; restored calcium and phosphorus concentrations; increased bone density, thickness, weight and tensile strength	Pandit et al. (2020)
	FA-modified liposomes	Ultrasonic crushing	Strong targeting; dual action of anti-osteosarcoma and bone protection	QUE	Inhibited the proliferation, migration and invasion of osteosarcoma cells and promoted their apoptosis; significantly inhibited tumor growth in vivo	Zwicke et al. (2012), Jing et al. (2022)
	(DSS)6 modification targeting liposomes	Membrane hydration method	Strong affinity for bone tissue; combining anti-aging and bone regeneration	QUE	Eliminated bone tissue senescent cells and increased the bone formation rate; upregulated osteogenic markers such as OCN and Runx2; increased bone trabecular volume and the number of calcified nodules in BMSCs	Xing et al. (2023)
	QUE microemulsion + caffeic acid phenethyl ester liposome-chitosan-poloxamer 407-poly-N-isopropylacrylamide hydrogel complexes	Water titration + composite assembly	“Anti-inflammatory-osteogenic” time-sequential release; synergistic effect of silver nanoparticles	QUE + caffeic acid phenethyl ester + silver nanoparticles	Reduced the M1-type macrophage ratio and ROS levels; increased ALP activity and OCN expression in PDLSCs; reduced RANKL; increased alveolar bone BV/TV and OPN-positive area; restored collagen fiber alignment	Zhang C. et al. (2025)
	Solid lipid nanoparticles β-TCP 3D scaffold complexes	Melt emulsification	Scaffold-carrier integration for targeted delivery and bone tissue growth guidance	QUE + vitamin D3	Promoted OB proliferation and differentiation and reduced osteosarcoma cell activity; reduced TRAP expression and resorption pit formation in OCs	Bose et al. (2024)
	Soybean phosphatidylcholine-modified nanolipid carrier-calcium phosphate scaffold complexes	Thermal homogenization + lyophilization	Drug-carrier-scaffold synergy; excellent biocompatibility	QUE for phosphorylation modification	Significantly promoted the formation of bone callus and reduced abnormal fibrous tissue; accelerated the healing of femoral defects and improved the quality of bone regeneration	Kamal et al. (2023)

### 3.5 Metal coordination modifications

Metallic materials typically possess excellent electrical and thermal conductivity, mechanical strength, and ductility, and are capable of forming stable nanoparticles (Coetzee et al., 2020). Metal compounds, especially metal oxides and MOFs, tend to have porous structures and high specific surface areas (Wang et al., 2024). Metals and their compounds commonly used in the medical field not only exhibit excellent chemical stability and can interact with human tissues while avoiding significant immune reactions, but also provide the necessary mechanical support for regenerative tissues due to their appropriate mechanical strength (Prasad et al., 2017). Additionally, some metallic materials can modulate cellular behavior and body metabolism, while also conferring the ability to deliver drugs precisely (Shabatina et al., 2023). Thus, the synergistic effect of metals and metal compounds can significantly improve the performance of QUE in terms of stability, bioavailability, and osseointegration, showing great promise for application in bone tissue regeneration.

It was found (Ferrer et al., 2006) that vanadium dichloride oxide is highly biocompatible and has good coordination ability. The complex formed by binding with QUE exhibits excellent stability, maintaining the drug’s activity for a long period in the organism and demonstrating significant therapeutic effects on bone regeneration. Specifically, this complex not only promoted the synthesis of Col I in OBs but also stimulated ERK phosphorylation in a dose-response manner. In addition, the reaction of QUE with strontium chloride under alkaline conditions results in the formation of a stable, water-soluble complex. Due to the high affinity of strontium ions for HAP, the complex was able to target and enrich the bone defect site. Experimental results showed that strontium chloride-QUE complexes enhanced ALP activity and mineralized nodule formation in OBs, exhibiting a high safety profile (cell survival >80% at 72 h) (Pimenta et al., 2025). Furthermore, ultra-small Fe-QUE nanocomplexes were prepared by wet chemistry. After ligand binding with anhydrous iron chloride, the stability, water solubility, and antioxidant properties of QUE were enhanced. In in vitro osteogenesis assays, the ultra-small iron-QUE nanocomplexes exhibited excellent free radical scavenging ability, promoted macrophage polarization to the M2 type, and reduced the release of pro-inflammatory cytokines TNF-α and IL-1β by inhibiting NF-κB pathway activation. Further application of these ultra-small iron-QUE nanocomplexes to arthritis model mice significantly reduced inflammation and fibroplasia, inhibited bone erosion and structural damage, while increasing BV/TV, BMD, and trabecular bone thickness (TB.Th) (Han et al., 2023). Building on this, loading ultra-small Fe-QUE nanocomplexes onto ROS-responsive injectable hydrogels (HA-modified 3-aminophenylboronic acid crosslinked with polyvinyl alcohol via borate ester bonding to form a porous network with ROS-responsive properties) could achieve precise drug release with both injectability and self-adaptability. Further incorporation of appropriate minocycline into this hydrogel could enable dual antibacterial-osteogenic drug slow release. *In vivo* experiments showed that this multifunctional drug delivery system significantly reduced inflammation and oxidative stress, decreased bone resorption by inhibiting RANKL/RANK signaling, and activated the BMP2 pathway to promote new bone formation. The expression of pro-inflammatory factors IL-1β and TNF-α decreased, while the expression of the anti-inflammatory factor IL-10 increased in periodontitis model rats after this treatment. Additionally, BV/TV and Tb.N were significantly increased, and the expression levels of Runx2, OCN, and OPG were also significantly elevated (Zhu et al., 2025).

Metal complexes alone have limitations, such as insufficient mechanical support and lack of drug carriers, but by combining them with nanoscaffolds, these issues are effectively addressed. Raj Preeth et al. (2021) encapsulated QUE-zinc chelates in PCL/Gel electrospun nanofiber scaffolds, which enhanced their biological stability and provided an ideal environment for cell attachment and growth. This scaffold not only significantly promoted ALP activity and calcium deposition in MG-63 cells but also up-regulated the expression of Runx2, ALP, and Col I, as well as stimulated angiogenesis, providing the necessary nutrient and oxygen supply for bone tissue repair and regeneration. Additionally, the chelation of QUE with magnesium-doped calcium silicate ceramic nanoparticles using the co-precipitation technique creates a stable environment for QUE, enhancing bone mineralization and cellular metabolism by releasing calcium and magnesium ions. This was then loaded onto PCL/Gel polymer electrospun scaffolds, further improving physical support and facilitating cell adhesion. Experimental data showed that this composite scaffold effectively promoted the proliferation and differentiation of OBs and supported the formation of new bone tissues in a denser and more solid manner (Preethi and Bellare, 2021). It is worth mentioning that Ou et al. (2024) combined QUE with copper-manganese bimetallic organic nanosheets (formed by self-assembly of porphyrin derivatives with copper and manganese ions, with dual functions of acoustic-dynamic antimicrobial and nano-enzymatic antioxidant) through liganding and further encapsulated them with the antimicrobial peptide Je-1 to form a hydrogel. The advantages of this composite system include good biocompatibility, high functional integration, and ultrasound-triggered precision treatment, which simultaneously addresses the three major problems of infection, inflammation, and bone regeneration. The results of *in vitro* and *in vivo* experiments have confirmed its efficacy: it not only significantly promotes ALP activity and mineralized nodule formation in BMSCs but also activates the JAK-STAT and Wnt signaling pathways to promote osteogenesis. It also promotes human umbilical vein endothelial cell migration and angiogenesis by activating the HIF-1α/VEGFA pathway. At the same time, this composite system can regulate macrophage polarization toward an anti-inflammatory phenotype, effectively reducing inflammation levels and improving the bone repair microenvironment. After treatment of rats with cranial bone defects in a model of combined methicillin-resistant *Staphylococcus aureus* infection, infection-related bone destruction was attenuated, and the amount of new bone formation was significantly increased and maturely mineralized.

The bone regeneration effect of QUE was significantly enhanced by the ligand modification of metal ions. This modification not only improved the stability and bioavailability of QUE but also greatly enhanced its anti-inflammatory and antioxidant activities, thereby promoting bone tissue repair more effectively. Additionally, the metal ions provided further bone regeneration-promoting effects and reduced the metabolic rate of QUE *in vivo*. To better illustrate the differences and merits of these delivery systems, their main features are compiled in Table 5. Metal-ligand modification significantly broadens the potential of QUE in the treatment of complex bone injuries and is expected to be an effective therapeutic strategy for bone regeneration in the future.

**TABLE 5:** Metal coordination systems for quercetin delivery in bone repair.

Types of drug delivery systems	Primary materials/Trim	Preparation method	Highlighting advantages	Drugs for loading	Main biological effects	References
Metal coordination modification	Vanadium dichloride complex	Solution synthesis method	Excellent biocompatibility; high coordination ability; excellent stability; long drug activity period	QUE	Promoted Col I synthesis in OB, stimulated ERK phosphorylation in a dose-response manner, and enhanced the activation of osteogenic differentiation signaling pathways	Ferrer et al. (2006)
	Strontium chloride complex	Solution synthesis method	Stable water solubility; targeted enrichment of bone defect sites	QUE	Enhanced ALP activity and mineralized nodule formation in OB; achieved a cell survival rate of >80% at 72h, demonstrating high safety	Pimenta et al. (2025)
	Ultra-small iron nanoparticles	Wet chemical method	Improved QUE stability, water solubility and antioxidant properties; excellent free radical scavenging ability	QUE	Scavenged free radicals and improved cell survival in inflammatory environments; promoted macrophage polarization to the M2-type; inhibited the NF-κB pathway; decreased TNF-α and IL-1β release; decreased bone erosion; increased BV/TV, BMD and Tb.Th	Han et al. (2023)
	Ultrasmall iron nanoparticles-ROS-responsive hydrogels	Wet chemical method + hydrogel crosslinking	ROS-responsive, injectable; realizes antimicrobial-osteogenic synergy	QUE + minocycline	Decreased IL-1β and TNF-α and elevated IL-10; inhibited the RANKL/RANK pathway to reduce bone resorption and activated the BMP-2 pathway to promote osteogenesis; increased BV/TV, Tb.N and the expression of Runx2, OCN, and OPG	Zhu et al. (2025)
	QUE-zinc chelate + PCL/gelatin scaffolds	Chelation reaction + electrospinning technology	Provides an environment for cell attachment; QUE is biostable	QUE	Promoted ALP activity and calcium deposition; up-regulated Runx2, ALP, and Col I expression; stimulated angiogenesis and improved the nutritional supply for bone repair	Raj Preeth et al. (2021)
​	Magnesium-doped calcium silicate ceramic nanoparticles + PCL/gelatin scaffolds	Co-precipitation technology + electrospinning technology	Calcium and magnesium ions enhance bone mineralization and cell metabolism; provide physical support and cell attachment environment	QUE	Promoted OB proliferation and differentiation, supported the dense formation of new bone tissue, and enhanced bone mineralization efficiency	Preethi and Bellare (2021)
	Copper-manganese bimetallic organic nanosheets + Je-1 hydrogel	Coordination self-assembly + solution reaction method	Combination of acoustic power antimicrobial and nano-enzymatic antioxidant functions; excellent biocompatibility	QUE	Activated the JAK-STAT and wnt pathways to promote osteogenesis; enhanced angiogenesis via the HIF-1α/VEGFA pathway; modulated macrophage anti-inflammatory polarization; attenuated infection-associated bone destruction and increased new bone formation and mineralization	Ou et al. (2024)

### 3.6 Nanomaterials

Nanomaterials are materials that measure 1–100 nm in at least one dimension. Due to their unique structure, nanomaterials exhibit physical, chemical, and biological properties that differ significantly from those of macroscopic materials, often including a high specific surface area, quantum effects, strong mechanical properties, and good biocompatibility (Joudeh and Linke, 2022). Additionally, the surfaces of nanomaterials can be functionalized and modified to obtain specific functionalities and selective recognition capabilities, allowing them to recognize and bind to specific cells or tissues (Sanità et al., 2020; Wietrzyk et al., 2025). These properties enable nanomaterials to offer significant advantages in drug delivery systems, facilitating the efficient delivery of drugs to specific body parts or cells, thereby enhancing efficacy, reducing side effects, and improving therapeutic precision (Yusuf et al., 2023). Therefore, the use of nanomaterials for QUE delivery is highly advantageous and provides a proven therapeutic option for the repair of bone defects.

Nanoparticles have nanoscale dimensions in three-dimensional orientation, which endows them with high surface energy and activity suitable for drug encapsulation and delivery. Among them, mesoporous bioactive glass nanoparticles (MBGNPs) exhibit multiple advantages, including high porosity, high specific surface area, and excellent immunocompatibility. MBGNPs encapsulated with appropriate concentrations of QUE significantly upregulated the expression of OPN and VEGF, enhanced calcium nodule formation and ALP activity in PDLSCs, and inhibited the inflammatory response in RAW264.7 cells. Moreover, QUE-loaded MBGNPs significantly increased bone mass and decreased the expression level of the inflammatory marker inducible nitric oxide synthase (iNOS) in alveolar bone defect model rats (Yang S. Y. et al., 2024). However, MBGNPs alone lacked adhesion, plasticity, and compressive strength, which was effectively optimized by the mixture of HA-sodium alginate hydrogel. In in vitro osteogenesis experiments, QUE-loaded MBGNPs/HA/sodium alginate nanocomposite pastes significantly enhanced the proliferation of BMSCs and upregulated osteogenesis-related proteins (Runx2, Col I, OCN, and OPN) and their gene expression (Sohrabi et al., 2024). Additionally, mesoporous silica nanoparticles can also serve as a drug delivery platform for QUE loading and delivery. On this basis, adding Col I can enhance osteogenic activity. Further introduction of PDA can endow the system with photothermal properties and pH responsiveness, enhancing its precise killing effect on tumor cells. The fabricated QUE-loaded Col I-PDA-mesoporous silica nanoparticles not only significantly increased the cell viability and ALP activity of BMSCs, but also up-regulated the expression of osteogenesis-related genes, such as Runx2, OPN, OCN, etc., promoted collagen secretion and mineralization of the extracellular matrix, and inhibited the proliferation and survival of osteosarcoma cells in nude mice *in vivo* (Shi et al., 2022). A new study (Sharma et al., 2025) pointed out that sodium selenite offers multiple advantages for drug delivery: not only does it provide micronutrients essential for maintaining bone health, but it also has good antioxidant properties and enhances the stability of the drug in solution. QUE can be adsorbed on the surface of selenium nanoparticles through noncovalent bonding, and its nanoscale size and surface charge can enhance cellular uptake. The cytotoxicity of QUE-selenium nanoparticles is low, with the cell survival rate still >80% at high concentrations (80 μM). Moreover, the effect was excellent in bone regeneration experiments, as it increased the BV/TV and Tb.N of drilled bone defect model mice and significantly increased the mineralized tissue in the bone defect area. However, the controllability and targeting of the drug encapsulated in nanoparticles alone are poor. Therefore, Zhang’s team (Zhang H. et al., 2025) utilized a one-pot method to control drug release by encapsulating QUE in methionine-modified carboxymethyl chitosan (CMCS) after loading it onto the MOF material ZIF-8, enhancing stability through hydrophobic interaction and responding to the ROS environment through oxidation of thioether bonds. The resulting methionine-modified CMCS@ZIF-8@QUE nanoparticles demonstrated excellent bone regeneration promotion *in vitro*, significantly scavenging ROS, down-regulating the expression of pro-inflammatory factors (IL-1β, TNF-α, and iNOS), promoting osteogenic differentiation of BMSCs, and increasing the expression of osteogenic genes (Col I, Runx2, and OCN). In addition, methionine-modified CMCS@ZIF-8@QUE nanoparticles reduced inflammatory cell infiltration and improved cartilage matrix integrity in osteoarthritis model rats, synchronizing the improvement of the infectious inflammatory microenvironment with bone repair.

In addition, two-dimensional nanoscale nanosheets and nanomembranes have significant research and application value in the biomedical field (Murali et al., 2021). In this regard, nanofiber membranes based on polylactic acid and poly (ethylene oxide) exhibit excellent biocompatibility, which can help QUE achieve precise and controlled drug release. Furthermore, this QUE-loaded nanofiber membrane can significantly inhibit the migration and proliferation of osteosarcoma cell lines MG-63, U-2OS, and SaOS-2, reduce their metabolic activities, and induce massive apoptosis while blocking the cell cycle (Hudecki et al., 2023). Additionally, the addition of MgO to PLGA nanofiber membranes helped maintain its bioactivity during QUE delivery and synergized its osteogenic effect, thereby improving the therapeutic outcome. The PLGA/MgO/QUE nanocomposite membrane, which had been multi-functionalized, could activate the Wnt/β-catenin pathway to promote osteogenesis, significantly promote the proliferation and migration of BMSCs, and up-regulate the expression of osteogenesis-related genes such as Runx2, Col I, OPN, ALP, and BMP2 (He et al., 2022). A recent study (Wu et al., 2023) demonstrated a porous nanosheet constructed from polyimide and molybdenum disulfide, which exhibited excellent mechanical, biocompatible and antimicrobial properties, strongly ensuring the safety of the drug delivery process. In the *in vitro* assessment of osteogenesis, the QUE-loaded molybdenum disulfide/polyimide nanosheets significantly enhanced the proliferation and ALP activity of BMSCs, and inhibited the proliferation, differentiation, and gene expression of OCs. Additionally, the nanosheets showed good osteointegration properties after implantation into bone defects in New Zealand white rabbits, which not only increased the amount of bone formation but also improved BMD, BV/TV, Tb.N, and Tb.Th in general. Notably, Cordoba et al. (2015) utilized covalent grafting to prepare QUE in a two-dimensional nanoscale form and formed a nanocoating on the surface of a titanium substrate to create a novel implant. The titanium implant functionalized with QUE nanocoatings showed excellent performance in promoting bone healing and improved the adhesion level and metabolic activity of BMSCs. In a further study (Cordoba et al., 2018), this QUE nano-coated titanium implant inhibited the formation of multinucleated TRAP-positive cells and down-regulated the expression of bone resorption-related genes such as TRAP, CTSK, MMP-9, and hydrogen ion ATPase. After implantation into tibial defects in New Zealand white rabbits, it significantly reduced OC activity and promoted the regeneration of bone defects.

The introduction of nanomaterials has broadened the role of QUE in bone regeneration. Nanomaterials not only effectively protect QUE from degradation by the *in vivo* environment but also regulate its release rate and improve the accuracy of its action at the target site. Additionally, the excellent surface properties of nanomaterials facilitate cell attachment and proliferation, thus promoting bone tissue repair. For quick reference and intuitive comparison, the key parameters of these strategies are summarized in Table 6. In the future, emphasis should be placed on optimizing the design of nanomaterials to further enhance the effectiveness of QUE in bone regeneration therapy. Moreover, the safety and efficacy of long-term use should be evaluated to ensure that QUE-loaded nanomaterials can effectively promote bone tissue repair, reduce the risk of complications, and provide better therapeutic outcomes for patients in a real clinical setting.

**TABLE 6:** Nanomaterial-based delivery systems for quercetin in bone therapy.

Types of drug delivery systems	Primary materials/Trim	Preparation method	Highlighting advantages	Drugs for loading	Main biological effects	References
Nanomaterials	MBGNPs	Sol-gel method	High porosity; high specific surface area; excellent immunocompatibility	QUE	Upregulated OPN and VEGF expression, enhanced calcium nodule formation and ALP activity in PDLSCs; inhibited the inflammatory response in RAW264.7 cells; increased bone mass and decreased iNOS levels	Yang S. Y. et al. (2024)
	MBGNPs/hyaluronic acid-sodium alginate nanocomposite paste	Sol-gel method	Excellent adhesion, plasticity and compressive strength	QUE	Enhanced BMSCs proliferation; upregulated the expression of Runx2, Col I, OCN, OPN and other osteogenic proteins and genes	Sohrabi et al. (2024)
	Collagen-PDA-mesoporous silica nanoparticles	Sol-gel and layer-by-layer self-assembly methods	pH-sensitive drug release; photothermal killing of tumor cells	QUE	Promoted proliferation, ALP activity and Runx2/OPN/OCN expression in BMSCs and enhanced extracellular matrix mineralization; inhibited the proliferation and survival of osteosarcoma cells	Shi et al. (2022)
	Selenium nanoparticles	Chemical reduction	Antioxidant; QUE stabilized; sized for cellular uptake	QUE	Exhibited low toxicity; increased BV/TV and Tb.N, and promoted mineralized tissue formation in bone defect areas	Sharma et al. (2025)
	Methionine-modified CMCS encapsulating ZIF-8 metal-organic frameworks	Self-assembly method	ROS environmentally responsive controlled release drugs; enhanced QUE stability	QUE	Scavenged ROS and down-regulated IL-1β/TNF-α/iNOS; promoted osteogenic differentiation and Col I/Runx2/OCN expression in BMSCs; reduced inflammatory infiltration and improved cartilage matrix integrity	Zhang H. et al. (2025)
	Polylactic acid-polyethylene oxide nanofiber membrane	Electrostatic spinning	Excellent biocompatibility; precise and controlled drug release	QUE	Inhibited the migration, proliferation and metabolic activity of osteosarcoma cells; blocked the cell cycle and induced apoptosis	Hudecki et al. (2023)
	PLGA/MgO/QUE nanocomposite membrane	Electrostatic spinning	QUE activity long-lasting; MgO synergistic osteogenesis	QUE	Promoted the proliferation and migration of BMSCs; up-regulated Runx2, Col I, OPN, ALP, and BMP2 gene expression; enhanced the activation of the Wnt/β-catenin pathway	He et al. (2022)
	Molybdenum disulfide/polyimide porous nanosheets	Powder mixing-press sintering + salt immersion method	Excellent mechanical properties; excellent biocompatibility; antimicrobial capacity	QUE	Enhanced the proliferation and ALP activity of BMSCs and inhibited the expression of OC-related genes; increased the amount of bone formation and improved the indexes of BMD, BV/TV, Tb.N, and Tb.Th	Wu et al. (2023)
	QUE nano-coated-Ti implants	Covalent grafting	High osseointegration capacity	QUE	Enhanced BMSCs adhesion and mineralization; inhibited the formation of multinucleated TRAP-positive cells; down-regulated bone resorption genes such as TRAP and CTSK; promoted bone defect regeneration	Cordoba et al. (2015), Cordoba et al. (2018)

### 3.7 Drug modification

Drug modification is the process of structurally modifying a drug molecule through chemical, physical, or biological means so that it binds to other substances or is encapsulated by a carrier (Juillerat-Jeanneret and Schmitt, 2007). This process plays an indispensable and crucial role in improving the effectiveness, safety, and applicability of drugs (Vargason et al., 2021). In modern medicine, many drugs with poor solubility, stability, short half-life, and high toxicity need to be chemically modified first to meet the evolving clinical therapeutic needs. In recent years, researchers have made significant progress in the therapeutic effects of bone regeneration by optimizing the drug properties of QUE through drug modification.

A previous study (Li Y. et al., 2024) showed that chitosan-QUE conjugates prepared by the carbodiimide reaction could fully utilize the synergistic effect. The chitosan-QUE conjugate not only effectively promoted calcium deposition and mineralization in BMSCs but also significantly enhanced the ALP activity of the cells and the gene expression of key osteogenic markers, such as Runx2 and Col I, while increasing the concentration of OCN and bone connexin. In addition, the conjugate reduced the expression levels of inflammatory factors such as TNF-α, IL-6, and IL-1β, effectively attenuating the LPS-induced inflammatory response. In further animal experiments, the chitosan-QUE conjugate still showed excellent osteogenic effects. It enhanced both bone healing and bone scab regeneration in zebrafish, an adult bone rupture injury model, and increased OB number and cell activity, while decreasing TRAP activity and hydroxyproline release. Moreover, the chitosan-QUE conjugate promoted angiogenesis in chick embryo vascularization experiments, helping to provide the necessary nutrient and oxygen supply during the regeneration and repair of bone tissue. Li X. et al. (2024) utilized the role of methotrexate in specifically activating macrophages at the site of inflammation, enabling the precise release of QUE. On this basis, the use of micelle wrapping guaranteed the stability of the drug during delivery, and the further connection of the MMP-responsive peptide to the micelle allowed it to accumulate in the inflammatory microenvironment. In the rheumatoid arthritis model rats treated with MMP-responsive peptide-methotrexate-micelles-QUE, serum levels of inflammatory factors such as IL-17, IL-6, and TNF-α were significantly reduced, and the damaged articular surfaces and cartilage structures were restored. During treatment, the MMP-responsive peptide-methotrexate-micelles-QUE reduced the expression levels of rheumatoid arthritis-related biomarkers CD44 and CD68, and no significant toxic side effects were observed. In a recent study, Luo and his colleagues (Luo et al., 2025) reacted QUE with phosphorus trichloride in a one-step process to generate p-QUE. p-QUE can self-assemble to form spherical nanoparticles with good aqueous solubility and colloidal stability due to the combination of a hydrophobic QUE core and hydrophilic phosphate groups. This method does not require complex coupling steps, and the process is simple and easy to scale up. It was shown that p-QUE could dose-dependently reduce the number of TRAP-positive OCs, down-regulate the transcription and expression of osteoblast-related genes (NFATc1, TRAP, CTSK), and block the RANKL-induced MAPK/phosphatidylinositol 3-kinase (PI3K) signaling pathway. In addition, p-QUE significantly increased BV/TV and Tb.N, reduced the number of TRAP-positive OCs in the bone marrow cavity, and inhibited the inflammatory response in the osteoporosis model of ovariectomized mice, confirming the same excellent therapeutic bone regeneration effects of p-QUE in pathological models.

Drug modification not only improves the stability of QUE and prevents its premature metabolism *in vivo* but also precisely regulates its release kinetics and enhances its accumulation at bone defect sites. Additionally, the unique physicochemical properties of drug modification can help promote the uptake and utilization of QUE by cells, thus accelerating the repair process of bone tissue. A systematic summary of the essential components and performance of these systems is presented in Table 7. Future studies should focus on optimizing drug modification strategies to maximize the effect of QUE in bone regeneration therapy. At the same time, the safety and tolerability of long-term application should be thoroughly investigated to ensure that QUE can stably promote bone tissue regeneration, reduce the occurrence of adverse effects, and achieve optimal patient recovery in clinical practice.

**TABLE 7:** Drug modification strategies for quercetin in bone therapy.

Types of drug delivery systems	Primary materials/Trim	Preparation method	Highlighting advantages	Drugs for loading	Main biological effects	References
Drug modification	Chitosan-QUE conjugate	Carbodiimide reaction coupling	Improved QUE stability and water solubility	QUE	Promoted calcium deposition, mineralization and ALP activity in BMSCs; upregulated osteogenic genes such as Runx2 and col I; reduced inflammatory factors such as TNF-α and IL-6; accelerated bone healing and increased the number of osteoblasts in zebrafish; promoted angiogenesis in chick embryos	Li Y. et al. (2024)
	MMP-responsive peptide-methotrexate multi-modified micelles	Membrane hydration method	Inflammatory microenvironment targeting; precise drug release	QUE	Reduced TNF-α, IL-6, IL-17 and ROS levels in RAW264.7 cells; reduced serum inflammatory factors in CIA rats, restored articular surfaces and cartilage structure; reduced rheumatoid arthritis markers (CD44, CD68) without causing significant toxic side effects	Li X. et al. (2024)
	p-QUE nanoparticles	One-step phosphorylation reaction + self-assembly method	Combination of hydrophobic core and hydrophilic phosphate groups; excellent water solubility and colloidal stability	QUE	Reduced TRAP-positive osteoclasts in a dose-dependent manner, downregulated osteoclast genes such as NFATc1 and CTSK, and blocked the RANKL-induced MAPK/PI3K/ROS pathway; increased bone volume fraction and Tb.N, and inhibited the M1-type inflammatory response in osteoporotic mice	Luo et al. (2025)

### 3.8 Discussion: comparison of quercetin delivery systems

**FIGURE 3:**
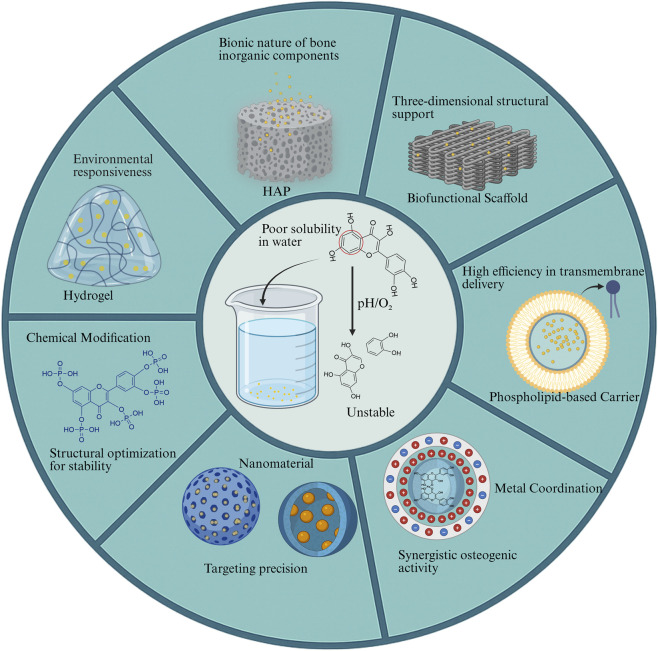
Core Concepts and Characteristics of Various Drug Delivery Systems. The chemical structure of QUE is non-polar, making it poorly soluble in water. Additionally, QUE has poor stability and is prone to degradation due to environmental factors such as pH and oxidative agents. Various drug delivery systems can enhance the therapeutic effects of QUE. Hydrogels can impart environmental responsiveness to QUE, while HAP provides bionic properties similar to bone inorganic components. Biodegradable scaffolds provide three-dimensional structural support for QUE, and phospholipid-based carriers enhance its transmembrane transport efficiency. Metal ion coordination can synergistically promote osteogenesis with QUE, while nanomaterials enable precise targeting. Chemical modifications can optimize the structure of QUE and improve its stability. Abbreviations: HAP: hydroxyapatite.

QUE’s wide variety of drug delivery systems are designed to enhance its physicochemical properties in all aspects, improve its pharmacokinetic properties, and endow it with precise targeting effects through a range of innovative strategies and advanced technological means. To further visualize the main properties of the seven types of quercetin delivery system materials, we have provided schematic diagrams (Figure 3). The application of these drug delivery systems can significantly enhance the value of QUE in clinical applications.

Although the seven types of delivery systems mentioned above can effectively improve the poor water solubility, rapid metabolism, and low stability of QUE, they differ significantly in functional characteristics, applicable scenarios, and underlying mechanisms. Hydrogels are the preferred carriers for local bone defect filling and sustained drug release due to their high water content, favorable biocompatibility, and controllable biodegradability. They enable stable long-term release of QUE and modulate the local immune microenvironment, but suffer from relatively low mechanical strength and are more suitable for non-weight-bearing regions. HAP shows high biosafety and excellent osteoconductivity owing to its chemical similarity to bone inorganic components, which effectively promotes mineral deposition and osteogenic gene expression. However, its drug-loading capacity and precise release control are relatively limited, making it more appropriate as a composite reinforcing material rather than an independent carrier. Biological functional scaffolds possess a distinct three-dimensional structure that mimics the extracellular matrix, supports cell infiltration and tissue ingrowth, and allows surface loading and sustained release of QUE. They exhibit outstanding advantages in large-segment bone defects and implant integration, but involve relatively complex fabrication processes. Phospholipid-based carriers significantly enhance the *in vivo* stability and targeting ability of QUE with high biocompatibility and encapsulation efficiency, especially suitable for systemic administration or minimally invasive local delivery. Nevertheless, they provide insufficient mechanical support. Metal coordination complexes endow materials with simultaneous antibacterial, antioxidant, and osteogenic activities via chelation between metal ions and QUE, showing great potential in infected bone defects. However, the potential long-term biosafety of released metal ions requires further attention. Nanomaterials achieve efficient loading and precise delivery of QUE due to their large specific surface area and modifiable surfaces, and their applications can be further expanded through surface functionalization. Nevertheless, the potential risks of metabolism and accumulation *in vivo* caused by the nanoscale size need to be carefully evaluated. Chemical modification of QUE optimizes its molecular structure to directly improve water solubility and targeting ability, and fundamentally resolves the intrinsic drawbacks of the drug. However, potential changes in the inherent pharmacological activity of QUE caused by chemical modifications should be rigorously evaluated for biosafety.

Collectively, hydrogels and HAP are mainly devoted to local bone microenvironment regulation and osteoconductive synergy; biological functional scaffolds and nanomaterials focus more on structural support and spatial guidance; phospholipid-based carriers, metal coordination complexes, and chemical modification strategies are dedicated to improving molecular stability and bioavailability of QUE. In practical applications, a single delivery system can hardly meet all the requirements for the repair of complex bone defects. In the future, it is necessary to develop multi-component composite platforms integrating the advantages of different systems, so as to achieve mechanical support, controlled drug release, immune regulation, and antibacterial functions simultaneously, and further promote the clinical translation of QUE in bone regeneration.

## 4 Summary and outlook

QUE, a naturally derived plant flavonoid with remarkable biological activities, exhibits promising application prospects in bone regeneration therapy by regulating the osteoblast-osteoclast balance, inhibiting inflammation and oxidative stress. However, its inherent drawbacks, including poor water solubility, rapid *in vivo* metabolism and low bioavailability, severely restrict its therapeutic value. This review systematically summarizes the research progress of seven major delivery systems, including hydrogels, HAP composites, biofunctional scaffolds, phospholipid carriers, metal coordination modification, nanomaterials and chemical modifications. These delivery systems significantly improve the local bioavailability and therapeutic efficacy of QUE by enhancing its solubility, increasing stability, retarding degradation rate, and achieving targeted delivery and controlled release. More importantly, these delivery platforms (especially hydrogels, scaffolds and composites) are often deeply integrated with bone tissue engineering, exerting multi-dimensional synergistic effects with QUE through three-dimensional scaffold support, biomimetic microenvironment construction and cell behavior regulation, thus strengthening the effect of bone regeneration and repair. *In vitro* and *in vivo* evidence demonstrates that QUE therapies based on optimized delivery systems exhibit superior osteogenic, anti-resorptive and anti-inflammatory activities compared with free QUE in models of osteoporosis, bone defects, osteoarthritis and osteomyelitis.

Although drug delivery systems have remarkably enhanced the application potential of QUE in bone regeneration, numerous challenges still remain. Most current studies focus on the construction of single carriers; future research should conduct in-depth investigations into the regulatory mechanisms of physicochemical properties of carriers on drug loading and release, targeting ability and biological effects, develop microenvironment-responsive intelligent delivery systems, and construct synergistic co-delivery systems co-loading QUE and multifunctional bioactive molecules for complex pathological bone defects, while promoting the large-scale and standardized preparation of superior carriers. The field still faces problems such as complex industrial processes, lack of quality control and poor batch-to-batch stability. In addition, insufficient data on long-term biocompatibility, degradation safety, *in vivo* metabolism and toxicology, as well as a critical lack of systematic clinical trial evidence for clinical translation, constitute core constraints for its clinical application. Therefore, intelligent carrier design, construction of multifunctional delivery systems, and systematic improvement of safety and clinical evaluation can effectively break through translational barriers and promote the early clinical application of QUE-based bone regeneration therapies.

In conclusion, the continuous optimization of drug delivery strategies, in-depth mechanistic studies, and the deep integration of QUE with advanced delivery technologies and bone tissue engineering concepts are expected to open new avenues for the development of efficient, safe and clinically feasible bone regeneration therapies, ultimately benefiting numerous patients with bone diseases.
